# *ortho*-Substituted 2-Phenyldihydroazulene Photoswitches: Enhancing the Lifetime of the Photoisomer by *ortho*-Aryl Interactions

**DOI:** 10.3390/molecules26216462

**Published:** 2021-10-26

**Authors:** Anna Ranzenigo, Franca M. Cordero, Martina Cacciarini, Mogens Brøndsted Nielsen

**Affiliations:** 1 Department of Chemistry, University of Florence, Via della Lastruccia 3-13, 50019 Sesto Fiorentino, Italy; anna.ranzenigo@unifi.it (A.R.); franca.cordero@unifi.it (F.M.C.); 2 Department of Chemistry, University of Copenhagen, Universitetsparken 5, 2100 Copenhagen Ø, Denmark

**Keywords:** cross-conjugation, electrocyclic reactions, linear conjugation, photochromism, positional isomerism

## Abstract

Photochromic molecules are systems that undergo a photoisomerization to high-energy isomers and are attractive for the storage of solar energy in a closed-energy cycle, for example, in molecular solar thermal energy storage systems. One challenge is to control the discharge time of the high-energy isomer. Here, we show that different substituents in the *ortho* position of a phenyl ring at C-2 of dihydroazulene (DHA-Ph) significantly increase the half-life of the metastable vinylheptafulvene (VHF-Ph) photoisomer; thus, the energy-releasing VHF-to-DHA back-reaction rises from minutes to days in comparison to the corresponding *para*- and *meta*-substituted systems. Systems with two photochromic DHA-Ph units connected by a diacetylene bridge either at the *para*, *meta* and *ortho* positions and corresponding to a linear or to a cross-conjugated pathway between the two photochromes are also presented. Here, the *ortho* substitution was found to compromise the switching properties. Thus, irradiation of *ortho*-bridged DHA-DHA resulted in degradation, probably due to the proximity of the different functional groups that can give rise to side-reactions.

## 1. Introduction

**Figure 1:**
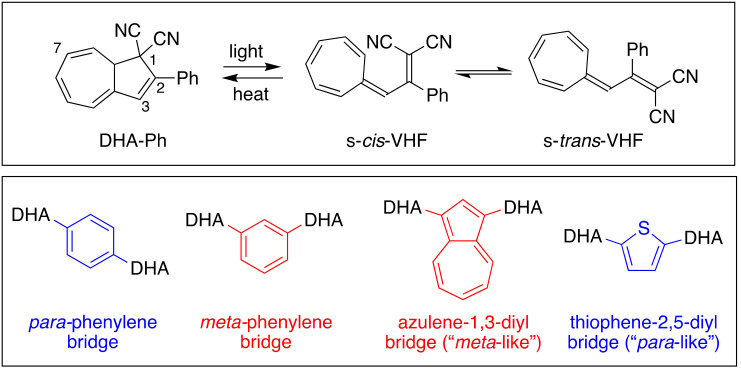
DHA-Ph/VHF-Ph monomer couple (top); previously reported *meta*- and *para*-connected DHA-based dimers (bottom).

**Figure 2:**
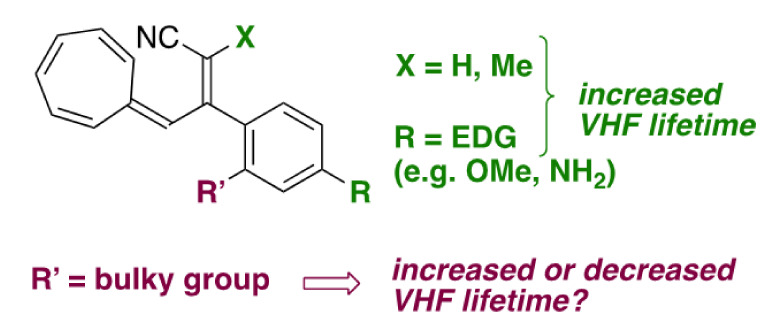
Introducing a hydrogen atom or methyl at the vinylic position of VHF (corresponding to position C-1 of DHA) or introducing an electron-donating group (EDG) at the *para* position of the phenyl enhances the VHF lifetime. Here, we investigate the influence of bulky groups at the *ortho* position of the phenyl substituent.

Organic photochromic systems are constituted by two isomeric species. Upon light irradiation, the low-energy isomer converts to the metastable high-energy isomer that can go back to the original isomer either thermally (*T-type* photoswitch) or photochemically (*P-type* photoswitch) [1,2,3,4]. Photochromic molecules have attracted increasing interest in recent years as candidates for storing solar energy in closed molecular systems, such as molecular solar thermal (MOST) systems, also termed solar thermal fuels (STF) [5,6,7,8,9]. One essential goal towards energy storage is the development of isomeric couples characterized by a high-energy isomer, which return to the original isomer within days or weeks, or whose thermal back-isomerization can be triggered upon demand, and which have high solar energy capture (quantum yield of photoisomerization). Among the different organic systems that have great potential as candidates for MOST, azobenzenes (AZB) and norbornadiene/quadricyclanes (NBD/QC) should be mentioned. Azobenzene photoswitches are characterized by *E/Z* isomerization of a N=N double bond and are robust systems with a broad absorption spectrum. Nevertheless, AZB-based compounds still need further improvements to be considered for MOST applications because of their lack of full photochemical conversion to the *Z* isomer, small *E/Z* absorption spectrum differentiation, and poor energy densities and quantum yields [10,11]. The NBD/QC pair was instead proposed for photochemical conversion and solar energy storage as far back as the 1980′s [12,13,14,15], but it was with the extensive studies of Moth-Poulsen and co-workers that it was developed and engineered up to laboratory-scale test devices [16]. This photochromic couple represents the most advanced organic system for MOST, although there are still challenges with this system to be solved. Another encouraging candidate for MOST is represented by the 2-phenyl-1,8a-dihydroazulene/vinylheptafulvene (DHA-Ph/VHF-Ph) couple (Figure 1, top box), which was first extensively studied by Daub and co-workers [17], and in the past decade by us [8]; this couple is the focus of this work. DHA-Ph has a characteristic absorption maximum at 360 nm in toluene and undergoes an electrocyclic light-induced ring-opening reaction to quantitatively form VHF-Ph (absorption onset 454 nm in toluene), with generally quite high quantum yield (60% in toluene). VHF-Ph reverts back to DHA-Ph by a thermally induced ring-closure or by the addition of Lewis acids [18]. The s-*trans* conformer of VHF-Ph is usually the most stable and the first step of the ring closure involves a change of conformation, from s-*trans* to s-*cis*, which is the reactive conformer for the cyclization [19]. To enable the use of DHA-Ph/VHF-Ph in advanced devices, the switching properties of the system need to be controlled. This aspect was fulfilled by modifying the parent structure with different functionalization on positions 1, 2, 3 and 7 of the original DHA-Ph scaffold (see numbering in Figure 1), which then drastically changes the half-life of the metastable VHF isomer [8]. For example, it has been shown that replacement of one cyano group in position 1 (corresponding to vinylic position of VHF, Figure 2) with a hydrogen atom, a methyl group or a thiazoline ring indefinitely halts the thermal ring closure [20,21]. Such modifications are particularly interesting in the context of MOST systems and long energy storage times.

Conversely, the replacement of one nitrile at C-1 (see DHA numbering, Figure 1) with a ketone, amide or ester group reduces the VHF half-life [22], making it generally difficult to establish a straightforward correlation between the electronic character of the functional group on C-1 and the thermal back-conversion. Instead, studying the VHF-to-DHA ring closure via the introduction of various substituents on positions 2, 3 and 7 allowed the establishing of linear-free energy relationships, i.e., Hammett correlations that reveal the dependency of the ring closure on the electron donor or electron acceptor nature of the substituent [23]. As a matter of fact, several aryl substituents were studied at C-2, and the electron-withdrawing character of a linearly conjugated *para*-substituent on the phenyl of DHA-Ph resulted in slightly faster thermal back-reaction than a cross-conjugated *meta* substituent (with half-lives of 108 and 85 min for *m*-CN and *p*-CN, respectively) [23]. On the contrary, electron-donating *para* substituents at the phenyl resulted in increased lifetimes of the VHF (with half-lives of 230 and 287 min for *p*-OMe and *p*-NH_2_, respectively; Figure 2) [23].

**Figure 3:**
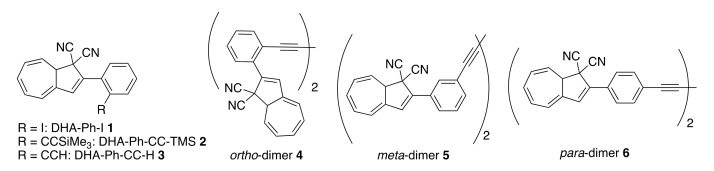
*ortho*-Phenyl-substituted DHAs and *ortho*-, *meta*- and *para*-linked DHA dimers.

Herein, we elucidate the effect of different substituents in the *ortho* position of the phenyl ring of DHA-Ph (compounds **1–3**, Figure 3), which so far have received very limited attention [24], and we compare their properties to the corresponding *meta*- and *para*-isomeric compounds. In fact, replacing the phenyl at C-2 with 9-anthryl was found to retard the VHF-to-DHA ring closure significantly [24], and preliminary data on an *ortho*-nitro substituted compound suggested that *ortho* substitution could be beneficial for retarding the back reaction [24]. Moreover, we know from other studies that if the s-*cis* conformer is promoted, then the VHF ring closure proceeds very fast [8]. Hence, we hypothesize that if we could disfavor the formation of the s-*cis* conformer in the s-*cis*/s-*trans* equilibrium by introducing unfavorable steric interactions enforced by the *ortho* substituent, then the lifetime of the VHF could be extended. A similar approach was previously reported for azobenzene derivatives [25,26,27], and sterical constraints were also found to play an important role for the NBD/QC couple [28]. Compounds **1–3** all contain an electron-withdrawing *ortho* substituent, and, in consequence, if steric factors are not important, then we should expect a faster VHF ring closure. On the contrary, if a slower VHF ring closure is actually observed, it would be a good indication that steric effects are in play that, accordingly, would more than counterbalance the electron-withdrawing effect of the substituent.

In addition, we present a study on dimeric molecules with two DHA-Ph photochromic units connected by *ortho, meta* or *para* diacetylene spacers. It was previously demonstrated that with multimode photoswitches constituted by two DHA units separated by a phenylene bridge, communication between the two units depends on the relative positions of the single units on the central benzene ring (*meta* or *para*, Figure 1, bottom box) [29]. The photoactivity of one DHA unit depends on whether its neighboring unit has already been converted to VHF or not, and a sequential switching between the three possible states (i.e., from DHA–DHA to DHA–VHF, and then to VHF–VHF) is achieved only with a *meta* connectivity [29,30]. The two sequential light-induced ring openings were explained by a significantly reduced photoactivity of DHA in the presence of a neighboring VHF electron acceptor unit (DHA–VHF). For a *para*-phenylene-bridged DHA dimer, we found that DHA ring opening is strongly inhibited and proceeds very slowly. The *para*-connected bridge separates the two DHA units by a linearly conjugated pathway, and the compound exhibits a redshifted absorption maximum in comparison to DHA-Ph and to the cross-conjugated *meta*-phenylene-bridged DHA dimer. Changes in the bridging unit from phenylene to thiophene-2,5-diyl (“*para*-like” connectivity, linear conjugation) or to azulene-1,3-diyl (“*meta*-like” connectivity, cross-conjugation) further confirmed that cross-conjugation allows sequential switchings, while linear conjugation reduces the photoactivity considerably, with full photoisomerization to VHF-VHF being accompanied by some degradation as well (Figure 1, bottom box) [29]. This “*meta*-rule” of photoactivity of phenylene-bridged photoswitch dimers was also established for azobenzenes [31,32], and is an important general design criterium. In this work, we introduced an acetylenic spacer to connect two DHA units via *ortho*, *meta* and *para* connectivities (compounds **4–6**, Figure 3), with the aim of shedding more light on the role of *ortho* connectivity.

## 2. Results and Discussion

### 2.1. Synthesis of ortho-Substituted DHA-Ph’s 1–4

**Scheme 1:**
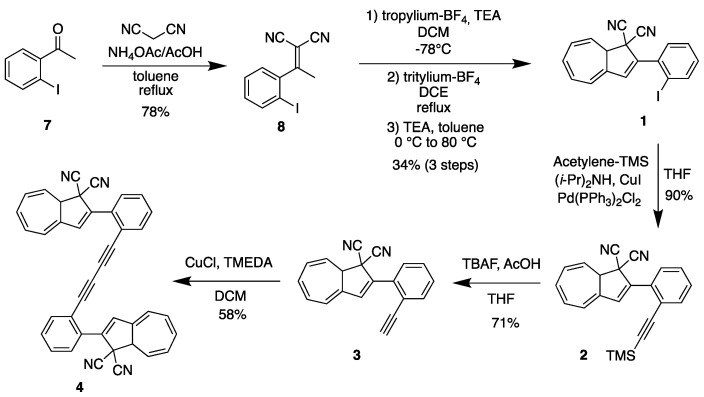
Synthesis of *ortho* derivatives **1–4**.

*Ortho*-substituted DHAs were synthesized following analogous procedures that were previously applied to *para*- and *meta*-substituted compounds (Scheme 1) [33]. The first step is a Knoevenagel condensation between *ortho*-iodoacetophenone (**7**) and malononitrile in toluene at reflux using AcOH and NH_4_OAc to give crotononitrile **8** in 78% yield. In the second step, crotononitrile **8** was treated with freshly prepared tropylium tetrafluoroborate at −78 °C in dichloromethane (DCM), and triethylamine (TEA) was slowly added. This step gave an alkylated intermediate that was used without purification for the next step. The crude reaction mixture was then dissolved in 1,2-dichloroethane (DCE) and heated to reflux in the presence of tritylium tetrafluoroborate for two hours. After being cooled to 0 °C and diluted with toluene, TEA was added over 20 min, and the intermediate VHF was then directly converted into DHA-Ph-I **1** (34% yield in 3 steps) by heating in the dark at 80 °C overnight. Further functionalization was subsequently achieved by Sonogashira couplings. Compound **1** was dissolved in THF at room temperature and (*i*-Pr)_2_NH, TMS-acetylene, Pd(PPh_3_)Cl_2_ and CuI were added to obtain DHA-Ph-CC-TMS **2** in 90% yield. Desilylation of **2** by the action of tetrabutylammonium fluoride and acetic acid in THF gave DHA-Ph-CC-H **3** in 71% yield. The terminal alkyne was finally used as a building block to synthetize *ortho*-dimer **4** (as a mixture of diastereomers on account of the stereocenter at C-8a of each DHA unit; i.e., a racemic mixture of enantiomers and a *meso* compound) by means of an oxidative Hay coupling with CuI and TMEDA in DCM under open air in 58% yield. The corresponding *meta*- and *para*-dimers **5** and **6** (Figure 3) were already synthetized in an analogous manner [34], but the UV-Vis absorption and switching properties were not investigated.

### 2.2. UV/Vis Absorption Spectroscopy and Switching Studies

**Figure 4:**
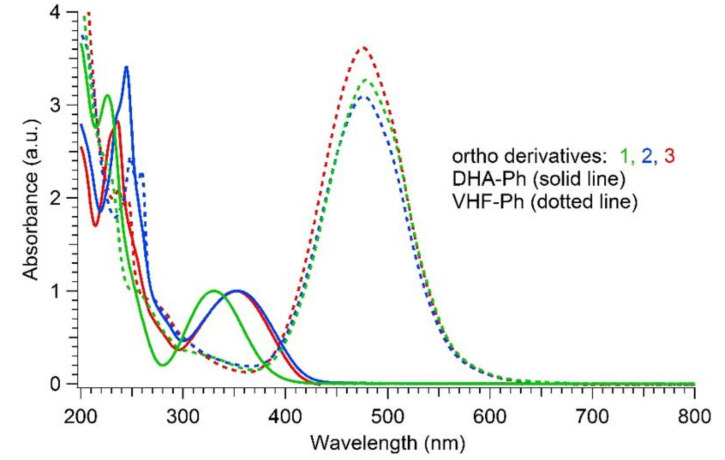
Normalized UV-Vis absorption spectra in MeCN of DHAs (solid line; longest-wavelength absorption maximum used for normalization) and VHFs (dotted line) for *ortho* compounds **1** (3.3 × 10^−5^ M, green), **2** (2.6 × 10^−5^ M, red) and **3** (1.6 × 10^−5^ M, blue).

The newly synthesized *ortho*-substituted DHAs **1–3** and their *meta* and *para* analogues (synthesized according to protocols given in the literature, [33,34]) were all photoactive and underwent thermally reversible isomerization (by irradiation at 365 nm) to their corresponding VHFs. Because of the very slow VHF-to-DHA transformation of the *ortho* derivatives, the thermal back-reactions (TBR) of the VHFs were evaluated at 35, 45 and 55 °C in MeCN, and the rates of the TBR were extrapolated from Arrhenius plots at 25 °C and listed in Table 1. The table also summarizes the characteristic absorption maxima of DHAs and VHFs in MeCN. As shown in Figure 4, the typical DHA absorption in *ortho*-DHA-Ph-I **1** was more than 20 nm blue-shifted in comparison to the corresponding *ortho*-DHA-Ph-CC-TMS **2** and DHA-Ph-CC-H **3**. Instead, for the *meta* and *para* series, no significant difference was depicted between the DHA and VHF forms of iodo- or alkynylated compounds.

**Figure 5:**
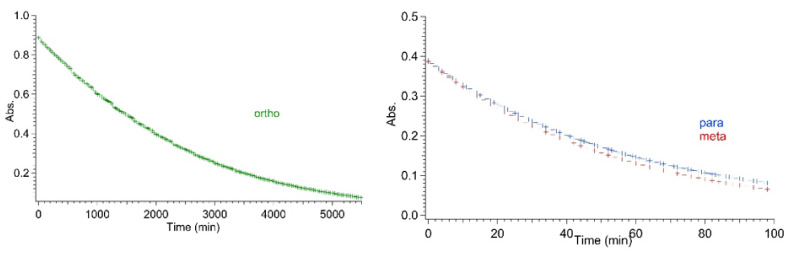
Comparison of VHF-Ph-I absorbance decay for *ortho* (left figure; green markers), *meta* and *para* (right figure; red and blue markers, respectively) derivatives at 35 °C.

As for the thermal back-reactions, all the *ortho* derivatives show a common general trend in that they always had a significantly longer VHF half-life in comparison to the corresponding *meta*- and the *para*-analogues, which conversely, were characterized by almost the same half-lives. While the thermal back-reaction of *meta* and *para* compounds ranged between 110 and 160 min irrespective of the substituent (iodo, TMS-ethynyl or ethynyl), in the case of *ortho*-connected derivatives, the presence of an ethynyl or TMS-ethynyl enhanced the thermal back-reaction by roughly a factor of 5 and 4, respectively, relative to an iodo substituent. Interestingly, the VHF of the *ortho*-iodo-substituted DHA **1** exhibited a half-life at 25 °C in acetonitrile, determined from the Arrhenius plot, that was 60-fold longer than the *meta* and *para* analogues. For comparison, Table 1 lists the half-lives for the *meta* and *para* iodo and alkynyl compounds extrapolated from Arrhenius plots, i.e., comparable conditions to those used for the *ortho* derivatives, as well as the values that were directly measured at 25 °C and those that were previously reported [23]. To visualize the great time lapse among the series and the significance of the *ortho* effect, the thermal decay for isomeric iodo-substituted VHFs at 35 °C is depicted in Figure 5. For the *ortho* compounds, a complete decay was achieved after more than 3 days at 35 °C, while it only took a couple of hours for the *meta* and *para* compounds.

It seems that the sterical influence of the *ortho* substituent played a significant role in the kinetics of the VHF ring closure. This could also be confirmed by the proportionality between the size of the substituent and the decrease in the half-life; for example, with the iodine that had a big atomic radius, we could see a huge impact on the half-life. For comparison, the parent VHF-Ph had a half-life of 218 min in MeCN at 25 °C [8]. Thus, for the *meta* and *para* compounds, the expected influence of an electron-withdrawing group can be seen, i.e., a faster ring closure reaction.

The quantum yields of photomerization of **1–3** were determined in acetonitrile; for **1**, it was determined up to 57%, quite similar to that reported previously for DHA-Ph (55%) [35]. The quantum yields of **2** and **3** were determined up to 59% and 67%, respectively, i.e., slightly higher than that of DHA-Ph (See Supplementary Materials for details).

**Figure 6:**
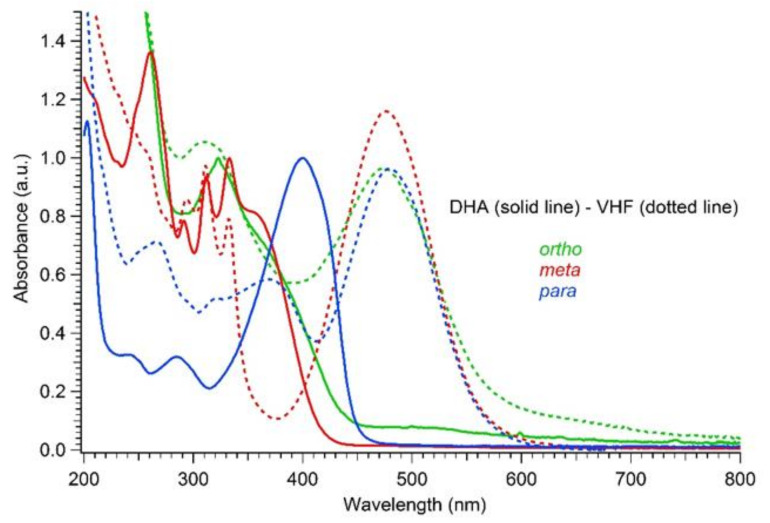
Normalized UV-Vis absorption spectra in MeCN of DHAs (solid line) and VHFs (dotted line) for *ortho*-dimer **4** (2.05 × 10^−5^ M, green), *meta*-dimer **5** (4.2 × 10^−5^ M, red) and *para*-dimer **6** (1.7 × 10^−5^ M, blue).

**Figure 7:**
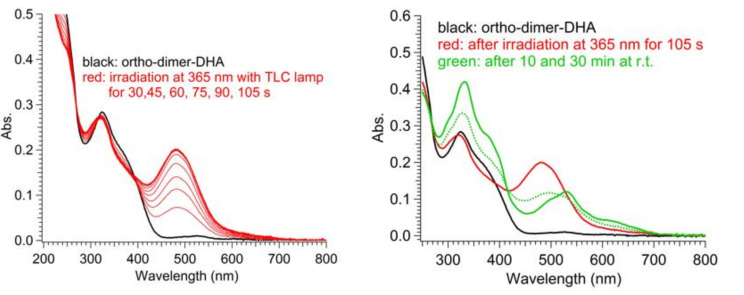
UV-Vis absorption spectra in MeCN of *ortho*-DHA-dimer **4.** Ring opening (left): before (black line) and after irradiation at 365 nm for 30–105 s (red lines). Ring closure (right): before irradiation (black line), after irradiation at 365 nm for 105 s (red line), after thermal relaxation for 10 min (dotted green line) and 30 min (solid green line) at r.t.

**Figure 8:**
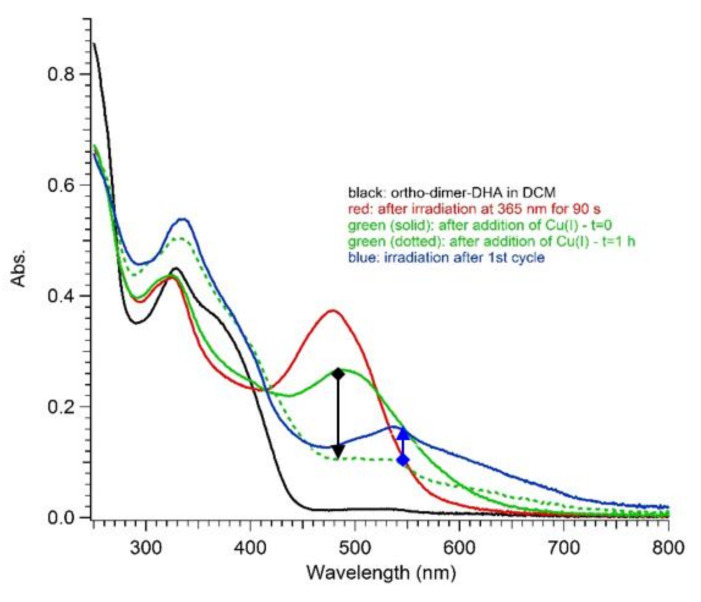
UV-Vis absorption spectra in DCM of *ortho*-DHA-dimer **4:** before irradiation (black line); after irradiation at 365 nm for 5–90 s (red lines); after addition of Cu(CH_3_CN)_4_BF_4_ at t = 0 (green solid line); one hour after addition of Cu(I) ions (green dotted line); and after irradiation at 365 nm to verify residues of activity (blue line). The black arrow highlights the thermal transformation and the blue arrow points out the photoactivity residue.

As for the three DHA-based dimers **4–6**, irradiation studies followed by UV-Vis absorption spectroscopy showed that the diacetylene spacers induced a similar trend as seen in the corresponding *para*- and *meta*-substituted phenylene-bridged photoswitch dimers. For *meta*-dimer **5**, irradiation for 5 min with a 365 nm LED lamp induced a complete disappearance of the characteristic shoulder at 365 nm, together with appearance of a redshifted maximum at 476 nm (solid and dotted red lines, Figure 6). Conversely, for *para*-dimer **6**, a consistent absorbance residue (dotted blue line, Figure 6) was maintained even after 7 min of irradiation owing to reduced photoactivity. The *ortho*-dimer **4** showed instead decomposition after only 2 min of irradiation with the LED lamp, while careful irradiation by TLC lamp (365 nm) allowed the detection of a clear isosbestic point between the graphs, a decrease in the DHA absorption, and the rising of a peak at 474 nm, which we attributed to a VHF-like species (Figure 6, solid and dotted green lines; Figure 7 (left), DHA-to-VHF ring opening of **4**). Within ten minutes after irradiation, the absorption at 474 nm decreased in intensity and red-shifted to a broad band at 530 nm. A recovery of the absorption at 365 nm that resembled the original spectrum, but with higher intensity, was detected (Figure 8, blue solid line). Nevertheless, a total loss of photoactivity was ascertained by further irradiation of the sample, meaning that decomposition or a competitive reaction other than the VHF-to-DHA transformation seems to have occurred.

To possibly reduce the extent of the undesired reaction, switching studies on **4** were also conducted in degassed dichloromethane, and the influence of the addition of Cu(I) ions, previously known to enhance the VHF-to-DHA conversion [18], was explored. Yet, as depicted in Figure 8, an analogous trend to that seen in acetonitrile was found in DCM, although a limited photoactivity of the sample was preserved and could be detected by further irradiation after the first light–heat cycle (see blue arrow, from green dotted line to blue solid line, Figure 8).

## 3. Discussion

New *ortho*-substituted 2-phenyl-DHAs were readily obtained from simple precursors, and subjecting the ethynyl-substituted derivative to an oxidative coupling provided a DHA dimer. Irradiation of this *ortho*-dimer gave a VHF-like UV-Vis absorption spectrum, but further decomposition of the sample could not be prevented even by triggering the thermal back-conversion with copper (I) ions. This behavior of the *ortho*-dimer contrasts that of related *meta*- and *para*-dimers, and it highlights something special with the *ortho* substitution pattern. A more specific influence of *ortho* substitution was, however, identified by studying DHA monomers. For these compounds, the *ortho* connectivity induced a strong retarding effect on the thermal ring-closure from VHF to DHA form, with half-lives being up to 60-fold longer in the case of iodo as a substituent. Thus, the half-life of the VHF was prolonged from 218 min to 5.4 days at 25 °C simply by introducing an *ortho*-iodo substituent on VHF-Ph. This is a remarkably simple way of tuning the VHF lifetime and is of particular interest in the design of MOST systems that aim to facilitate long energy storage times. We speculate that the enhanced lifetime of an *ortho* substituent may be due to a reluctance of the VHF to take the s-*cis*-VHF conformation that is required for the ring closure reaction and tentatively attribute the effect to the bulkiness of the substituent. Further studies are planned on the effect of bulkier substituents or of *ortho*-disubstituted phenyl rings.

## 4. Materials and Methods

Reactions requiring anhydrous conditions were carried out under a nitrogen atmosphere, and solvents were dried appropriately before use. All handling of photochromic compounds was conducted in the dark, with flasks and columns wrapped in aluminum foil. Thin-layer chromatography (TLC) was carried out on commercially available precoated plates (Silica 60). Spectrophotometric measurements were carried out in a 1-cm path length cuvette at 25 °C, unless otherwise stated. Spectrophotometric analysis of the ring-opening reaction was conducted by irradiating a solution of DHA (concentration range: 10^−5^ M) in the cuvette using a Thorlabs LED M365L2 for 365 nm. The thermal back-reaction was studied by heating the cuvette with the solution in a Peltier unit in the UV-Vis spectrophotometer. NMR spectra were acquired on a 500 MHz Bruker instrument equipped with a direct cryoprobe or a 500 MHz Varian spectrometer equipped with a direct broad-band probe. All chemical shift values in the ^1^H and ^13^C NMR spectra were referenced to the residual solvent peak (CDCl_3_ δ_H_ = 7.26 ppm, δ_C_ = 77.16 ppm). High Resolution Mass spectrometry (HRMS) was performed using either Electrospray Ionization (ESI) or Matrix Assisted Laser Desorption Ionization (MALDI), using a FT-ICR (Fourier Transform Ion Cyclotron Resonance) instrument. The quantum yield for the photoisomerization of **1–3** was measured using a high concentration regime (absorbance above 2 at wavelength of irradiation) with potassium ferrioxalate/tris(1,10-phenanthroline) as a chemical actinometer, following a general procedure [36]. Compounds **1–3** were isolated as racemic mixtures, while compound **4** was isolated as a mixture of diastereomers (racemic mixture of enantiomers and meso compound).

Synthesis of **8.** Iodo-acetophenone **7** (1 g, 4.1 mmol) and malononitrile (798 mg, 11.48 mmol) were dissolved in toluene (14 mL). NH_4_OAc (1.09 g, 13.94 mmol), dissolved in AcOH (1.61 mL), was added, the flask was equipped with a Dean–Stark apparatus and the reaction mixture was heated to reflux and stirred for 7 h. After cooling at room temperature, the reaction mixture was diluted with diethyl ether (10 mL), washed with water (10 × 20 mL) and brine (20 mL), and dried with MgSO_4_. Evaporation of the solvents resulted in product **8** as a pale, yellow solid. Recrystallization from boiling heptane (70 mL) gave the product (937 mg, 78%) as colourless crystals. HRMS ESI (C_11_H_7_IN_2_) calc. *m/z* = 316.95516 [M + Na]^+^, found 316.95485 [M + Na]^+^. ^1^H NMR (500 MHz, CDCl_3_): δ 7.94 (dd, *J* = 7.9, 1.1 Hz, 1H), 7.48 (td, *J* = 7.6, 1.1 Hz 1H), 7.17 (td, *J* = 7.7, 1.6 Hz 1H), 7.13 (dd, *J* = 7.7, 1.6 Hz 1H), 2.58 (s, 3H) ppm. ^13^C NMR (125 MHz, CDCl_3_): δ 179.3, 141.9, 140.2, 131.7, 129.0, 127.0, 111.7, 111.2, 92.8, 90.0, 25.3 ppm. Mp 118–119 °C.

Synthesis of **1.** Tropylium tetrafluoroborate (581 mg, 3.26 mmol), shredded with mortar and pestle, and crotononitrile **8** (790 mg, 2.67 mmol) were suspended in dry CH_2_Cl_2_ (36 mL) under argon atmosphere. The reaction mixture was cooled to −78 °C and Et_3_N (0.42 mL, 82.5 mmol) was added dropwise over 10 min. The solution was stirred for 20 min, and aqueous 2 M HCl (1 mL) was added. The organic phase was washed with water (2 × 10 mL) and dried with MgSO_4_. Evaporation of the solvents gave the nucleophilic addition product as orange crystals, and it was used for the next step without further purification. The crude mixture (970 mg, 2.52 mmol) and tritylium tetrafluoroborate (915 mg, 2.77 mmol) were dissolved in dichloroethane (17 mL) under argon atmosphere. The reaction mixture was stirred at reflux for 2 h (dark red solution), after which it was diluted with toluene (8.4 mL) and cooled to 0 °C. Et_3_N (0.5 mL, 3.63 mmol) was added over 20 min. The reaction mixture was then excluded from light and stirred at 80 °C for 3h and at 40 °C overnight. The solvents were evaporated in vacuo. Purification by flash column chromatography (SiO_2_, heptane, heptane/DCM 10:1–6:1–2:1–1:1–2:1) furnished the product **1** as an orange solid. Recrystallization from DCM/heptane gave a sample of pure **1** (423 mg, 34%) as orange crystals. HRMS ESI (C_18_H_11_IN_2_), calc. *m/z* = 383.00452 [M+H^+^], found 383.00417 [M+H^+^]. ^1^H NMR (500 MHz, CDCl_3_): δ 8.0 (dd, *J* = 8.0, 0.9 Hz, 1H), 7.64 (dd, *J* = 7.7, 1.4 Hz, 1H), 7.47 (td, *J* = 7.6, 1.1 Hz, 1H), 7.15 (td, *J* = 7.9, 1.6 Hz, 1H), 6.64–6.57 (m, 2H), 6.53 (dd, *J* = 11.2, 6.0 Hz, 1H), 6.37 (d, *J* = 5.9 Hz, 1H), 6.33 (ddd, *J* = 10.0, 6.0, 2.1 Hz, 1H), 5.81 (dd, *J* = 10.1, 3.7, 1H), 3.74 (dt, *J* = 3.7, 1.7 Hz, 1H) ppm. ^13^C NMR (126 MHz, CDCl_3_): δ 141.2, 140.4, 139.5, 137.9, 136.7, 131.4, 131.4, 130.9, 129.2, 128.5, 127.8, 121.7, 119.8, 114.7, 112.3, 100.3, 50.8, 48.9 ppm. Mp 110–111 °C.

Synthesis of **2.** DHA **1** (770 mg, 2.01 mmol) was dissolved in dry degassed THF (20 mL), and degassed (*i*-Pr)_2_NH (1.13 mL, 8.08 mmol), TMS-acetylene (1.15 mL, 8.08 mmol), Pd(PPh_3_)Cl_2_ (71 mg, 01 mmol) and CuI (8 mg, 0,04 mmol) were added under Ar atmosphere. The reaction mixture was stirred at room temperature overnight. The solvent was removed under reduced pressure, and the crude extract was purified via flash silica gel column chromatography (Eluent heptane/DCM 1.5:1). The product (640 mg, 90%) was obtained as an orange solid. HRMS ESI (C_23_H_20_N_2_Si) calc. *m/z* = 375.12934 [M + Na]^+^, found 375.13120 [M + Na]^+^. ^1^H NMR (500 MHz, CDCl_3_): δ 7.82 (dd, *J* = 8.0, 0.7 Hz, 1H), 7.62 (dd, *J* = 7.7, 1.1 Hz, 1H), 7.50 (s, 1H), 7.46 (td, *J* = 7.18, 1.4 Hz, 1H), 7.36 (td, *J*=7.6, 1,2 Hz, 1H), 6.57 (dd, *J* = 11.3, 6.2 Hz, 1H), 6.48 (dd, *J* = 11.3, 6.1 Hz, 1H), 6.34–6.28 (m, 2H), 5.81 (dd, *J* = 10.3, 3.7 Hz, 1H), 3.79 (dt, *J* = 3.8, 2.0 Hz, 1H), 0.23 (s, 9H) ppm. ^13^C NMR (126 MHz, CDCl_3_): δ 139.1, 137.8, 137.7, 134.9, 132.6, 131.1, 130.9, 129.1 (2C), 127.8, 127.1, 122.7, 121.4, 120.0, 115.3, 112.9, 104.3, 100.9, 50.9, 47.4, -0.14 (3C) ppm. Mp 114–115 °C.

Synthesis of **3.** To a stirred solution of **2** (500 mg, 1.42 mmol) in THF (109 mL) was added to AcOH (0.16 mL, 2.84 mmol) and a solution of tetrabutylammonium fluoride (1 M in THF; 1.42 mL, 1.42 mmol). The reaction mixture was stirred at rt for 2.5 h. The resulting dark yellow solution was diluted with Et_2_O (60 mL), washed with water (3 × 30 mL) and brine (30 mL), dried with MgSO_4_, filtered, and concentrated in vacuo. Purification by flash silica chromatography (DCM/heptane 1:1.5) gave the product (284 mg, 71%) as an orange solid. HRMS ESI (C_20_H_12_N_2_) calc. *m/z* = 303.08982 [M+H^+^], found 303.08934 [M + H]^+^. ^1^H NMR (500 MHz, CDCl_3_): δ 7.83 (dd, *J*=7.9, 1.3 Hz, 1H), 7.66 (dd, *J*=7.7, 1.3 Hz, 1H), 7.49 (td, *J*=7.7, 1.3 Hz, 1H), 7.39 (td, *J*=7.6, 1.3 Hz, 1H), 7.35 (s, 1H), 6.58 (dd, *J*=11.3, 6.2 Hz, 1H), 6.49 (dd, *J*=11.3, 6.2 Hz, 1H), 6.36 (dd, *J*=6.2, 1.7 Hz, 1H), 6.31 (ddd, *J*=10.3, 6.2, 2.0 Hz, 1H), 5.79 (dd, *J* = 10.2, 3.8 Hz, 1H), 3.78 (dt, *J* = 3.9, 2.0 Hz, 1H), 3.32 (s, 1H) ppm. ^13^C NMR (126 MHz, CDCl_3_): δ 138.7, 137.8, 137.4, 135.3, 132.9, 131.1, 131.0, 130.9, 129.4, 129.3, 129.3, 127.7, 127.2, 119.9, 115.2, 112.7, 83.0, 82.5, 50.9, 47.5 ppm. Mp 89.5–90 °C.

Synthesis of **4**. To a stirred solution of **3** (100 mg, 0.36 mmol) in DCM dry (50 mL) was added to TMEDA (42 mg, 0.36 mmol), CuCl (71 mg, 0.72 mmol) and 4 Å molecular sieves. The reaction mixture was stirred at rt with the open flask overnight. The resulting orange solution was filtered through Celite and the solvent was evaporated under reduced pressure. Purification by flash silica chromatography (toluene) gave the product (58 mg, 58%) as an orange solid. HRMS MALDI (C_40_H_22_N_4_) calc. *m/z* = 559.19227 [M+H^+^], found 559.19169 [M + H]^+^. ^1^H NMR (500 MHz, CDCl_3_): δ 7.85 (dd, *J*=7.6, 1.9 Hz, 2H), 7.69 (dd, *J*=7.8, 1.3, 2H), 7.53 (td, *J* = 7.8, 1.4 Hz, 2H), 7.41 (td, *J* = 7.6, 1.8 Hz, 2H), 7.36 (s, 1H), 7.34 (s, 1H), 6.55 (dd, *J* = 11.3, 6.3 Hz, 2H), 6.49 (dd, *J*=11.2, 6.0 Hz, 2H), 6.37 (d, *J* = 6.2 Hz, 2H), 6.31 (ddd, *J* = 10.2, 6.0, 2.1 Hz, 2H), 5.80 (dd, *J* = 10.1, 3.4 Hz, 2H), 3.79 (dt, *J* = 4.8, 2.4 Hz, 2H) ppm. ^13^C NMR (126 MHz, CD_2_Cl_2_): δ 139.1, 138.2, 137.5, 136.2, 134.0, 131.6, 131.3, 130.3, 129.7, 128.1, 127.8, 122.6, 121.4, 120.3, 115.6, 113.2, 82.6, 79.1, 51.3, 47.8 ppm. Mp 227.3–228 °C.

## Figures and Tables

**Table 1:** Characteristic longest-wavelength absorptions l_max_ (DHA value/VHF value) and half-lives of the thermal back-reaction VHF-to-DHA of *ortho*, *meta* and *para* analogues in MeCN extrapolated by Arrhenius plot at 25 °C unless otherwise stated.

	DHA-Ph-I	DHA-Ph-CC-TMS	DHA-Ph-CC-H	Dimer-DHA
*ortho* l_max_ (nm)	331/477	354/477	352/475	323/474
*ortho* t_1/2_ (min)	7.8 × 10^3^	2.0 × 10^3^	1.5 × 10^3^	decomp.
*meta* l_max_(nm)	356/478	357/476	355/476	333/476
*meta* t_1/2_ (min)	137 (157 ^a^)	160	160 (181 ^a^)	5 ^b^
*para* l_max_(nm)	360/476	365/478	361/477	400/480
*para* t_1/2_ (min)	110 (152 ^a^)	137	140 (137 ^a^)	4 ^b^

^a^ Literature data; measured at 25 °C [23]. ^b^ Measured at 55 °C.

## Data Availability

Spectral data can be found in the Supplementary Materials.

## Supplementary Materials

The following are available online. Figure S1: 1H-NMR spectrum of 8 in CDCl3 (500 MHz). Figure S2: COSY (left) and (right) 1H/13C HSQC spectra of 8 in CDCl3 (500/126 MHz). Figure S3: 13C spectrum of 8 in CDCl3 (126 MHz). Figure S4: 1H-NMR spectrum of 1 in CDCl3 (500 MHz). Figure S5: COSY (left) and (right) 1H/13C HSQC spectra of 1 in CDCl3 (500/126 MHz). Figure S6: 13C spectrum of 1 in CDCl3 (126 MHz). Figure S7: 1H-NMR spectrum of 2 in CDCl3 (500 MHz). Figure S8: COSY (left) and (right) 1H/13C HSQC spectra of 2 in CDCl3 (500/126 MH. Figure S9: 13C spectrum of 2 in CDCl3 (126 MHz). Figure S10: 1H-NMR spectrum of 3 in CDCl3 (500 MHz). Figure S11: COSY spectrum of 3 in CDCl3 (500 MHz). Figure S12: 13C spectrum of 3 in CDCl3 (126 MHz). Figure S13: 1H-NMR spectrum of 4 in CDCl3 (500 MHz). Figure S14: COSY spectrum of 4 in CDCl3 (500 MHz). Figure S15: 13C spectrum of 4 in CD2Cl2 (126 MHz). Figure S16: Left: Exponential decay of absorbance at 477 nm of 1VHF to 1DHA in acetonitrile at 35 °C (t_1/2_ = 1851 min). Right: Spectral evolution during thermal back- reaction of 1 in acetonitrile at 35 °C. Figure S17: Left: Exponential decay of absorbance at 476 nm of 1VHF to 1DHA in acetonitrile at 45 °C (t_1/2_ = 559 min). Right: Spectral evolution during thermal back- reaction of 1 in acetonitrile at 45 °C. Figure S18: Left: Exponential decay of absorbance at 476 nm of 1VHF to 1DHA in acetonitrile at 55 °C (t_1/2_ = 132 min). Right: Spectral evolution during thermal back- reaction of 1 in acetonitrile at 55 °C. Figure S19: Arrhenius plot for the 1VHF to 1DHA conversion. Figure S20: Arrhenius plot for the meta-VHF-Ph-I to meta-DHA-Ph-I conversion. Figure S21: Arrhenius plot for the para-VHF-Ph-I to para-DHA-Ph-I conversion. Figure S22: Left: Exponential decay of absorbance at 476 nm of 2VHF to 2DHA in acetonitrile at 35 °C (t_1/2_ = 492 min). Right: Spectral evolution during thermal back- reaction of 2 in acetonitrile at 35 °C. Figure S23: Left: Exponential decay of absorbance at 475 nm of 2VHF to 2DHA in acetonitrile at 45 °C (t_1/2_ = 142 min). Right: Spectral evolution during thermal back- reaction of 2 in acetonitrile at 45 °C. Figure S24: Left: Exponential decay of absorbance at 475 nm of 2VHF to 2DHA in acetonitrile at 55 °C (t_1/2_ = 46 min). Right: Spectral evolution during thermal back- reaction of 2 in acetonitrile at 55 °C. Figure S25: Arrhenius plot for the 2VHF to 2DHA conversion. Figure S26: Arrhenius plot for the meta-VHF-Ph-CC-TMS to meta-DHA-Ph-CC-TMS conversion. Figure S27: Arrhenius plot for the para-VHF-CC-TMS to para-DHA-Ph-CC-TMS conversion. Figure S28: Left: Exponential decay of absorbance at 475 nm of 3VHF to 3DHA in acetonitrile at 35 °C (t_1/2_ = 422 min). Right: Spectral evolution during thermal back- reaction of 3 in acetonitrile at 35 °C. Figure S29: Left: Exponential decay of absorbance at 475 nm of 3VHF to 3DHA in acetonitrile at 45 °C (t_1/2_ = 126 min). Right: Spectral evolution during thermal back- reaction of 3 in acetonitrile at 45 °C. Figure S30: Left: Exponential decay of absorbance at 474 nm of 3VHF to 3DHA in acetonitrile at 55 °C (t_1/2_ = 39 min). Right: Spectral evolution during thermal back- reaction of 3 in acetonitrile at 55 °C. Figure S31: Arrhenius plot for the 3VHF to 3DHA conversion. Figure S32: Arrhenius plot for the meta-VHF-Ph-CC-H to meta-DHA-Ph-CC-H conversion. Figure S33: Arrhenius plot for the para-VHF-Ph-CC-H to para-DHA-Ph-CC-H conversion. Figure S34: UV-Vis absorption spectra in MeCN of DHAs (solid line) and VHFs (dotted line) for ortho compounds 1 (3.3 × 10^−5^ M, green), 2 (2.6 × 10^−5^ M, red) and 3 (1.6 × 10^−5^ M, blue). Figure S35: Spectral evolution during ring-opening of 4 in acetonitrile at 25 °C. Figure S36: Left: Spectral evolution during thermal back- reaction of 5 in acetonitrile at 45 °C. Right: Exponential decay of absorbance at 474 nm of 5VHF to 5DHA in acetonitrile at 45 °C. Figure S37: Left: Spectral evolution during thermal back- reaction of 6 in acetonitrile at 55 °C. Top right: Exponential decay of absorbance at 474 nm of 6VHF to 6DHA in acetonitrile at 55 °C. Figure S38: Absorbance of ferrioxalate at 510 nm. Figure S39. Absorbance of 1VHF (first sample) vs. irradiation time during irradiation at 365 nm. Figure S40. Absorbance of 1VHF (second sample) vs. irradiation time during irradiation at 365 nm. Figure S41. Absorbance of 2VHF (second sample) vs. irradiation time during irradiation at 365 nm. Figure S42. Absorbance of 2VHF (second sample) vs. irradiation time during irradiation at 365 nm. Figure S43. Absorbance of 3VHF (second sample) vs. irradiation time during irradiation at 365 nm. Figure S44. Absorbance of 3VHF (second sample) vs. irradiation time during irradiation at 365 nm.

## Sample Availability

Samples of the compounds **1** and **4**. are available from the authors. Samples **2**, **3** and **5**, **6** are not available.
