# Supplementary material for: ortho-Substituted 2-Phenyldihydroazulene Photoswitches: Enhancing the Lifetime of the Photoisomer by ortho-Aryl Interactions
Source: Molecules. 2021 Oct 26;26(21):6462. doi: 10.3390/molecules26216462 (PMC8588411; doi:10.3390/molecules26216462)
Supplement: Supplementary file 1 [file molecules-26-06462-s001.zip › molecules-1417791-supplementary.pdf]

Supporting information

ortho-Substituted 2-phenyldihydroazulene photoswitches: enhancing the lifetime of the photoisomer by *ortho*-aryl interactions

Anna Ranzenigo[1], Franca Maria Cordero,[1] Martina Cacciarini[1,*] and Mogens Brøndsted Nielsen[2,*]

[1] Department of Chemistry, University of Florence, Via della Lastruccia 3-13, 50019 Sesto Fiorentino (FI), Italy;
[2] Department of Chemistry, University of Copenhagen, Universitetsparken 5, DK-2100 Copenhagen Ø, Denmark;
* Correspondence: martina.cacciarini@unifi.it; mbn@chem.ku.dk

TABLE OF CONTENTS

NMR spectra 2

UV-Vis absorption spectroscopy and switching studies 12

Quantum Yield 19

NMR spectra

Compound **8**

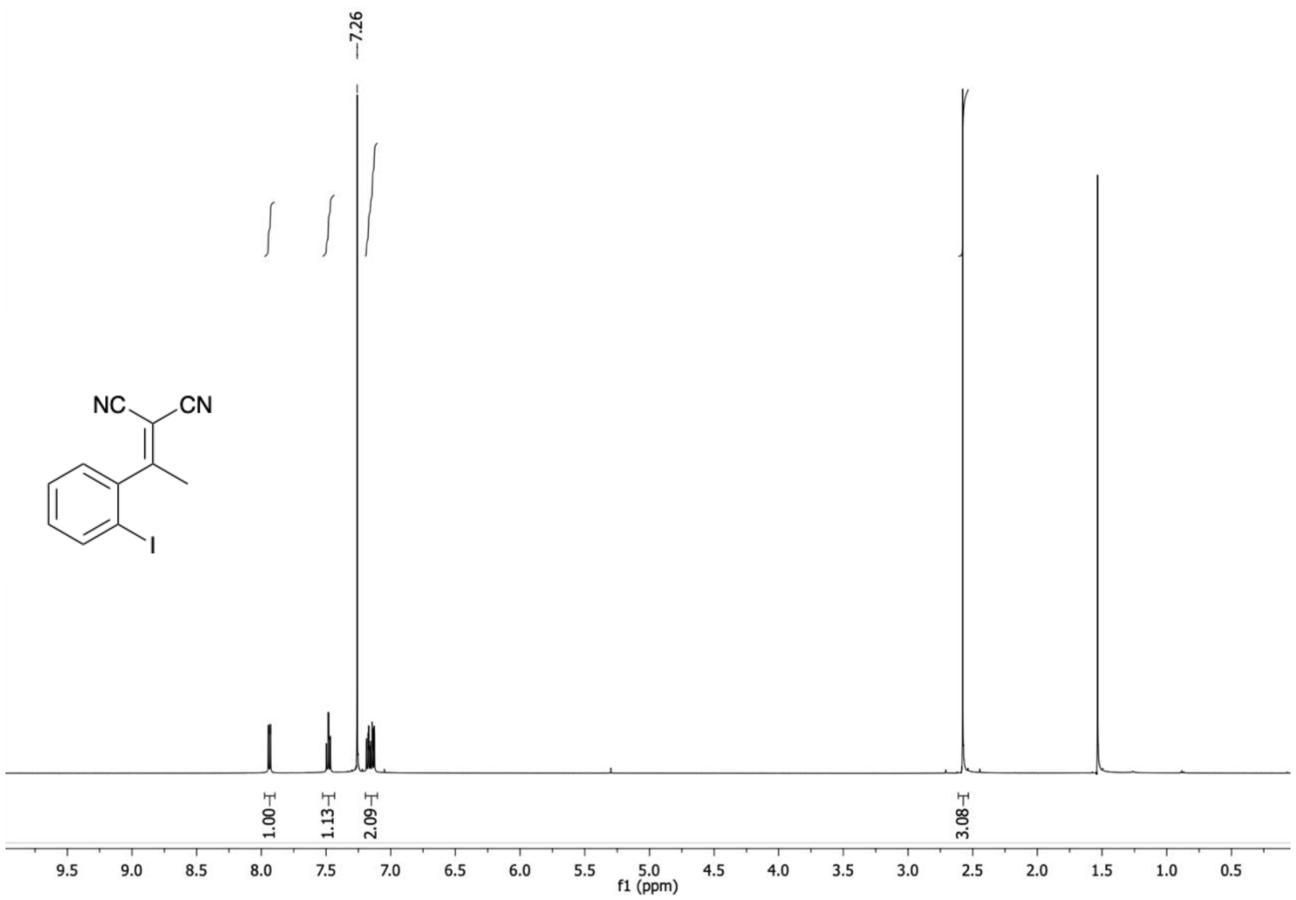

Figure S1: ^{1}H-NMR spectrum of **8** in $CDCl_3$ (500 MHz).

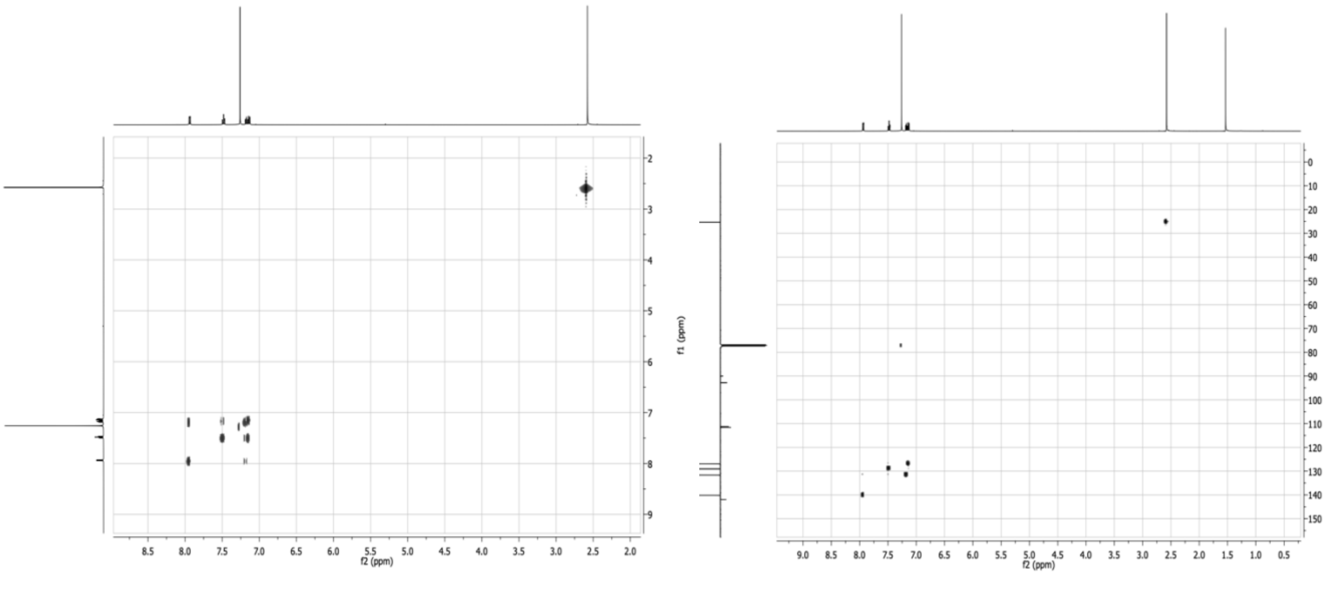

Figure S2: COSY (left) and (right) ^{1}H /^{13}C HSQC spectra of **8** in $CDCl_3$ (500 / 126 MHz).

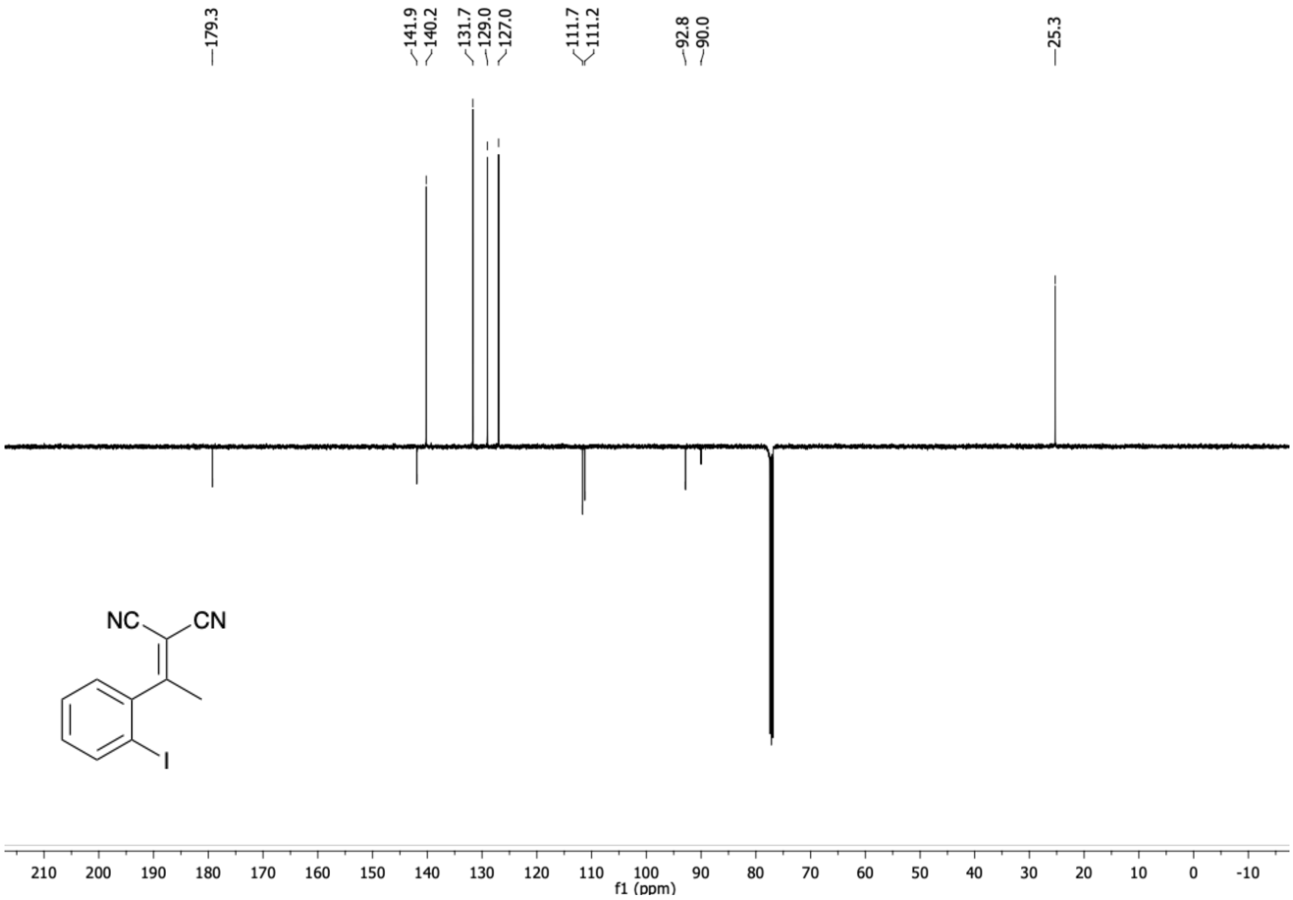

Figure S3: ^{13}C spectrum of **8** in $CDCl_3$ (126 MHz).

Compound **1**

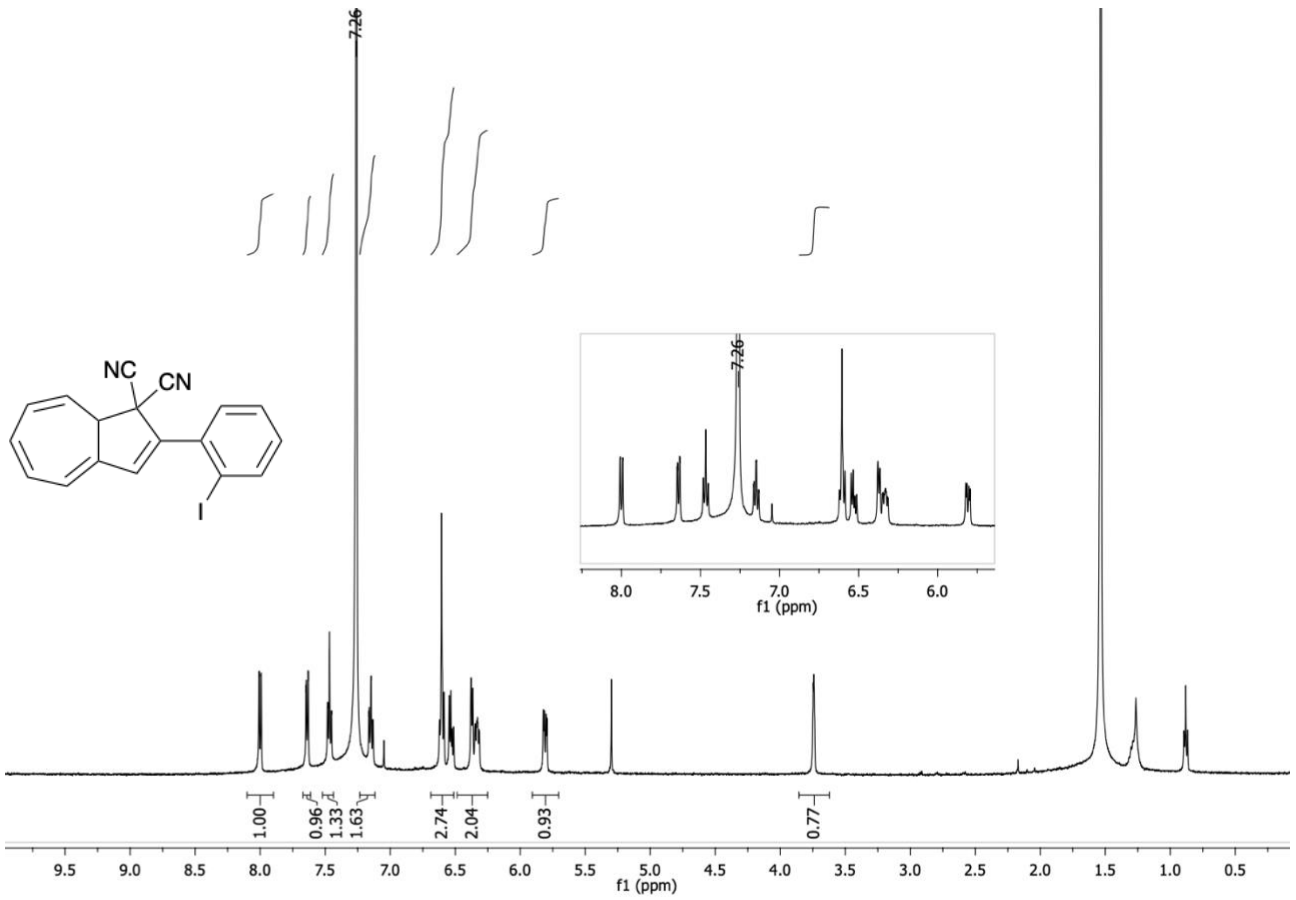

Figure S4: ^{1}H-NMR spectrum of **1** in $CDCl_3$ (500 MHz).

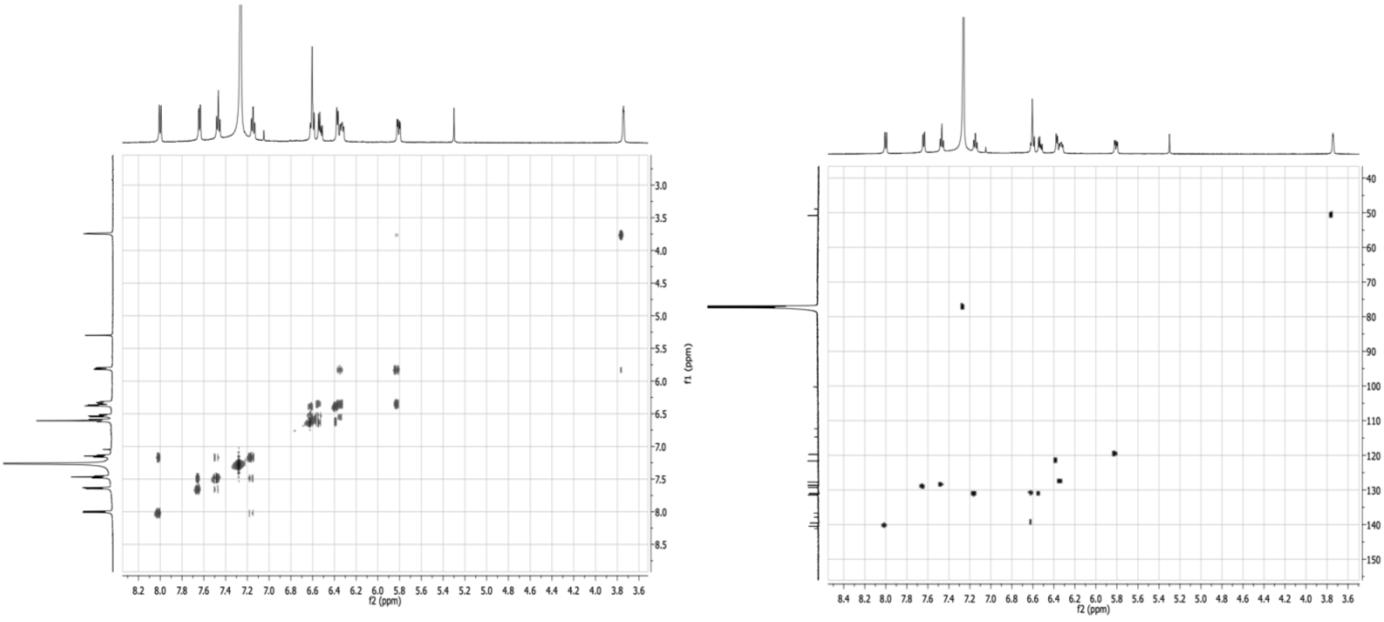

Figure S5: COSY (left) and (right) ^{1}H /^{13}C HSQC spectra of **1** in $CDCl_3$ (500 / 126 MHz).

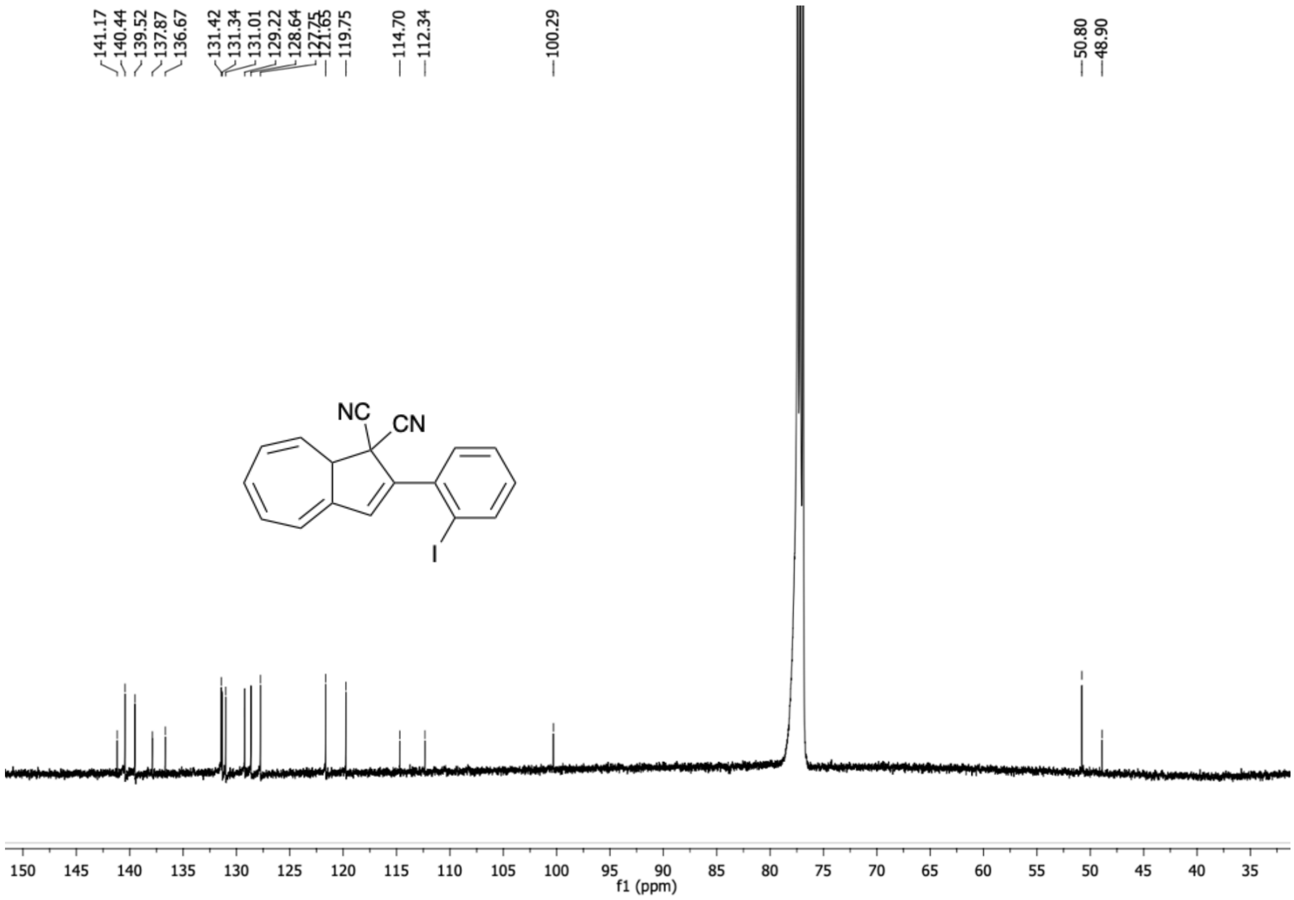

Figure S6: ^{13}C spectrum of **1** in $CDCl_3$ (126 MHz).

Compound **2**

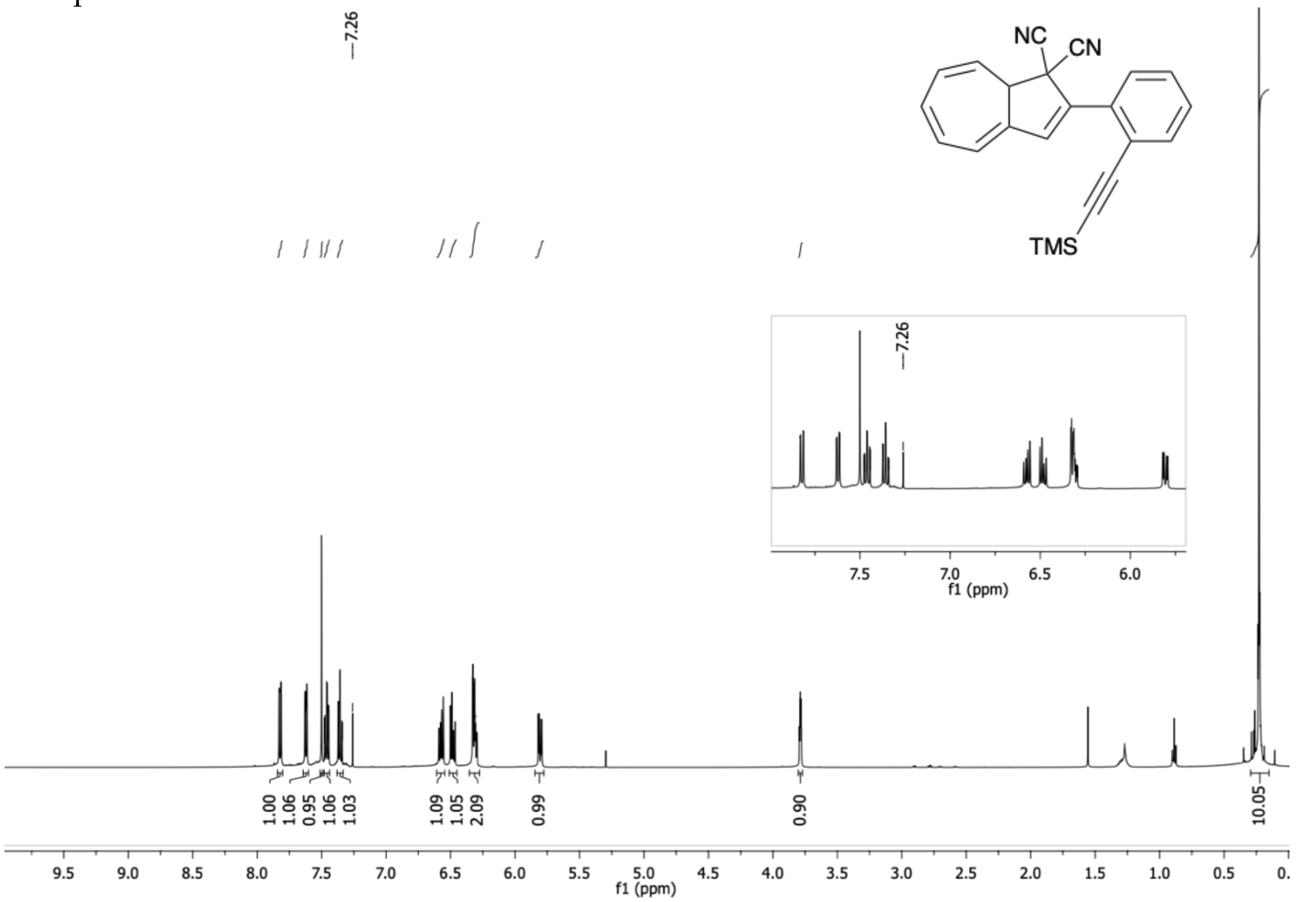

Figure S7: ^{1}H-NMR spectrum of **2** in $CDCl_3$ (500 MHz).

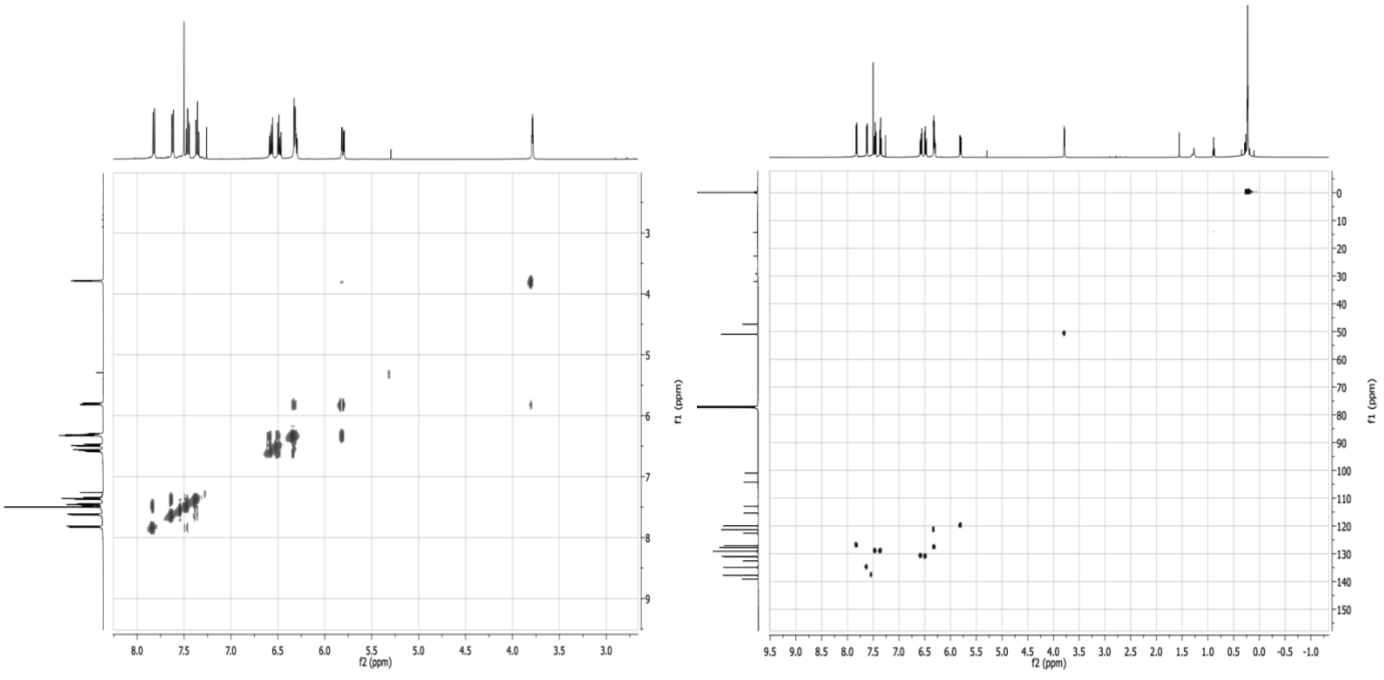

Figure S8: COSY (left) and (right) ^{1}H /^{13}C HSQC spectra of **2** in $CDCl_3$ (500 / 126 MHz).

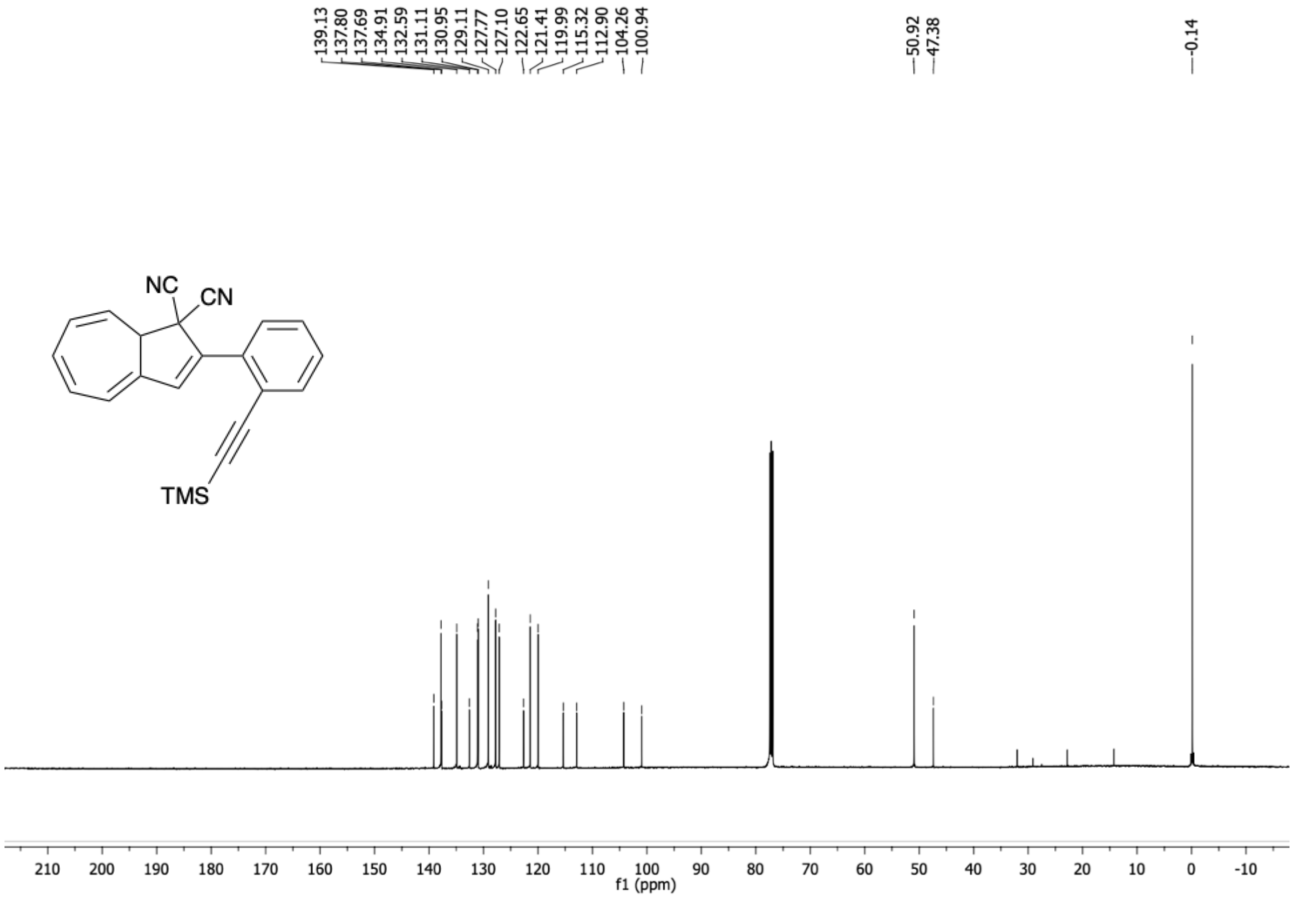

Figure S9: ^{13}C spectrum of **2** in $CDCl_3$ (126 MHz).

Compound **3**

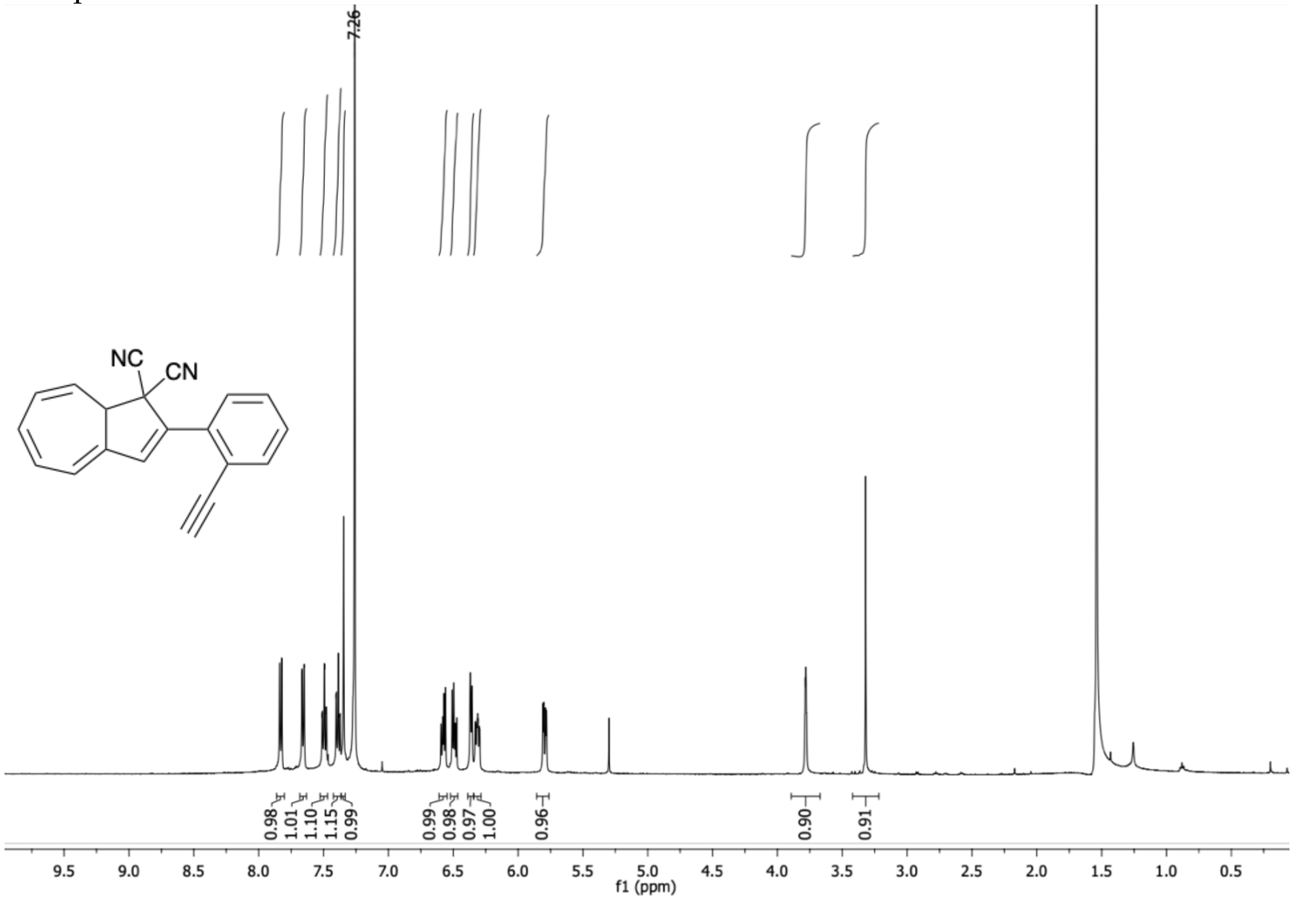

Figure S10: ^{1}H-NMR spectrum of **3** in $CDCl_3$ (500 MHz).

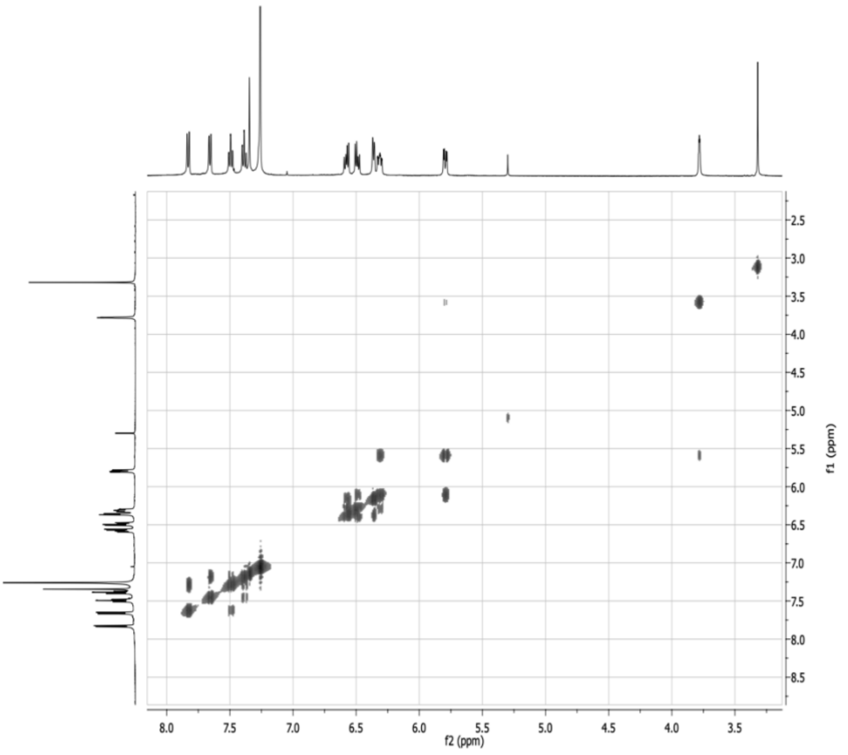

Figure S11: COSY spectrum of **3** in $CDCl_3$ (500 MHz).

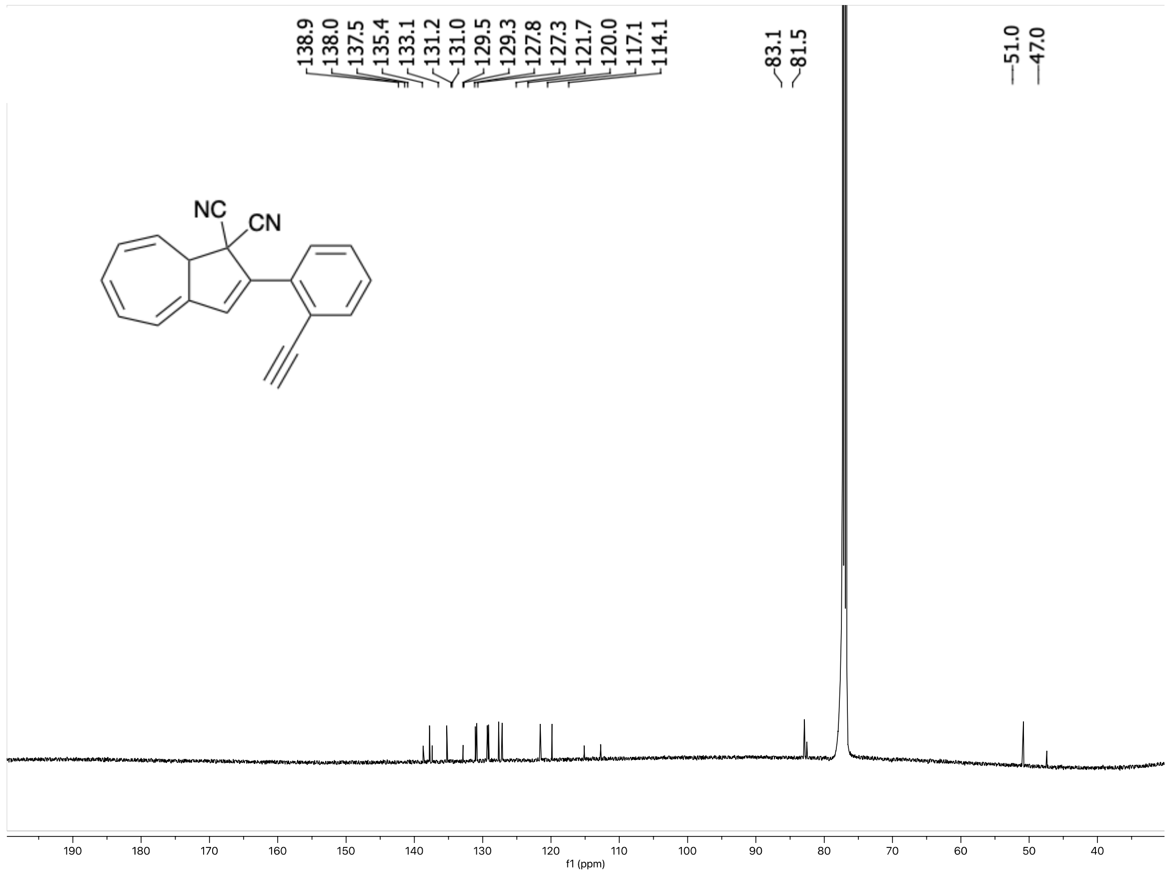

Figure S12: ^{13}C spectrum of **3** in $CDCl_3$ (126 MHz).

Compound **4**

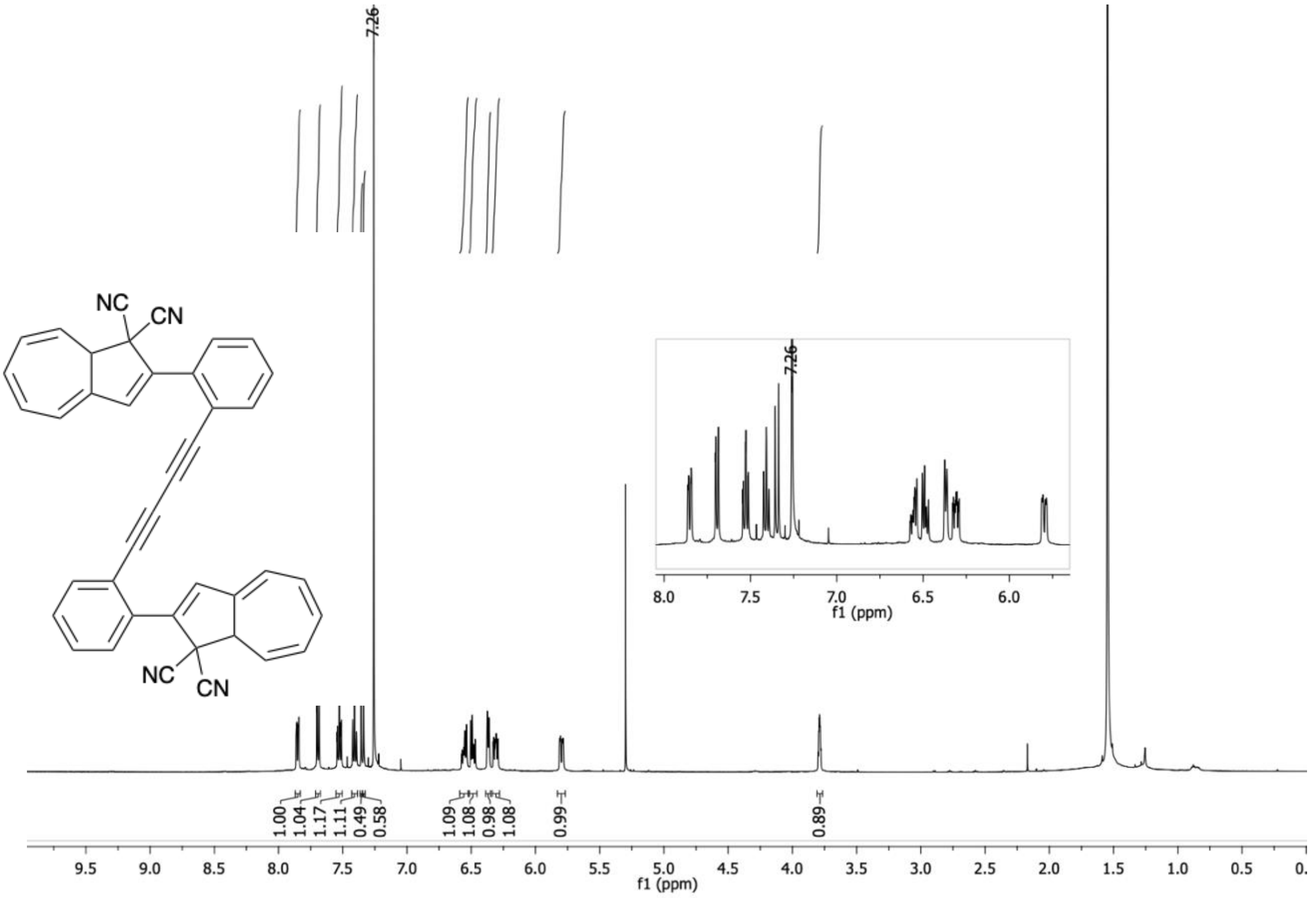

Figure S13: ^{1}H-NMR spectrum of **4** in $CDCl_3$ (500 MHz).

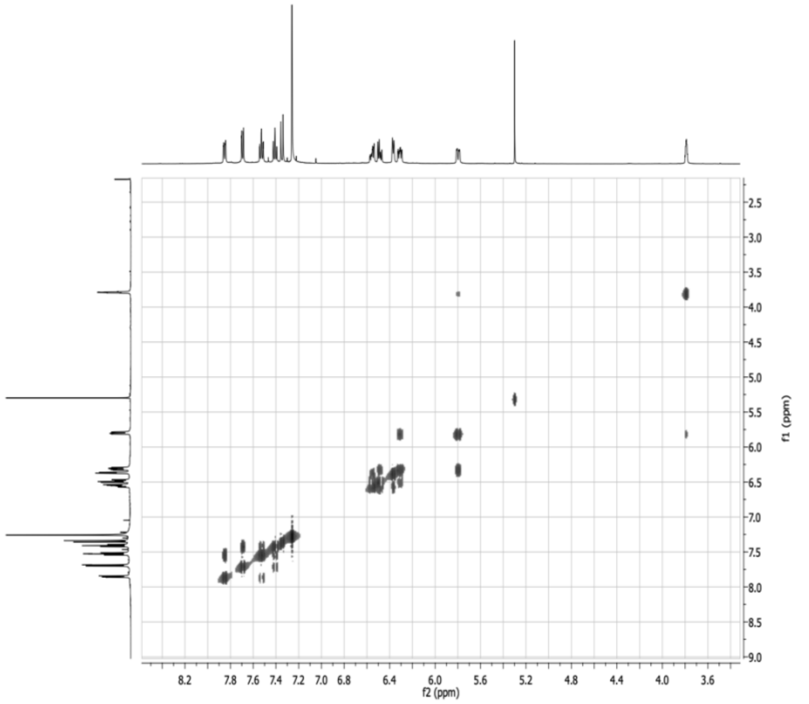

Figure S14: COSY spectrum of **4** in $CDCl_3$ (500 MHz).

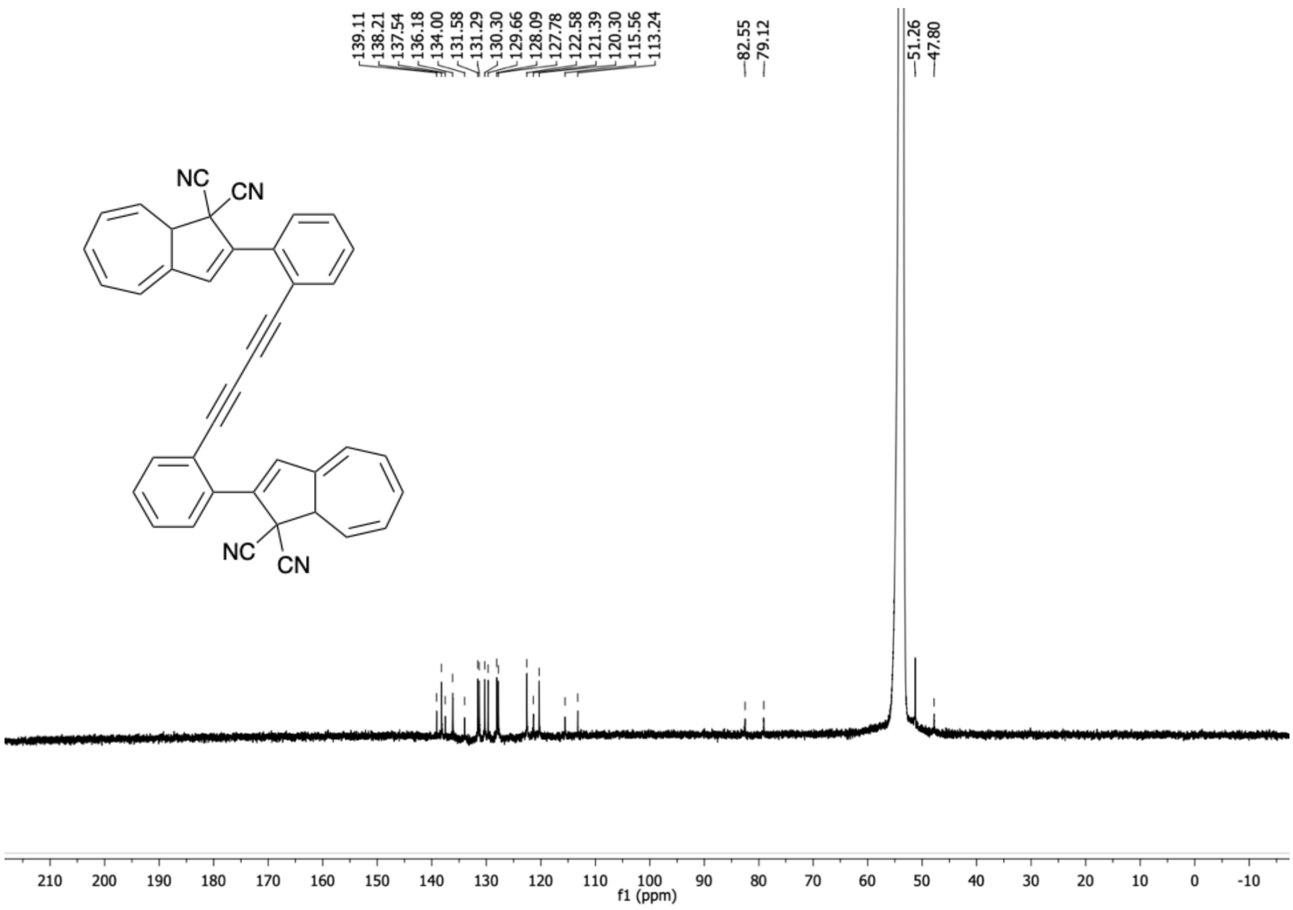

Figure S15: ^{13}C spectrum of **4** in CD_2Cl_2 (126 MHz).

UV-Vis absorption spectroscopy and switching studies

Compound **1**

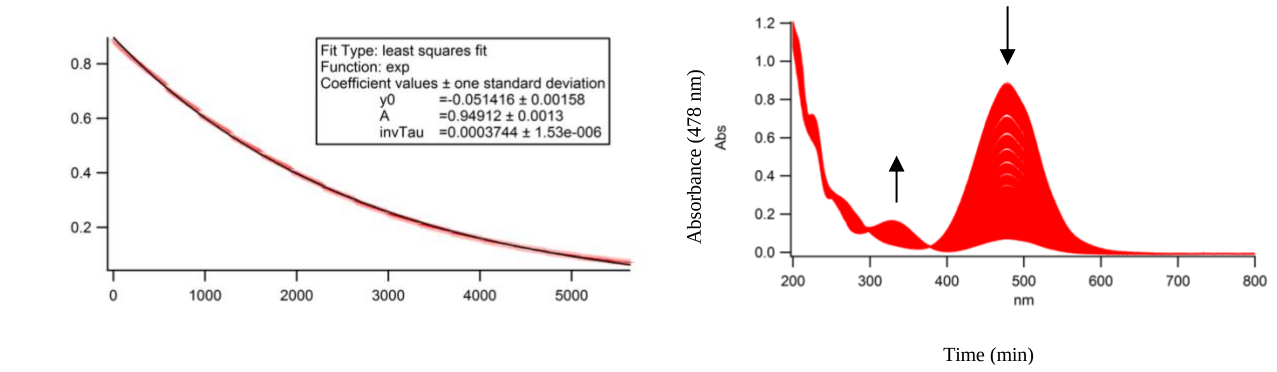

Figure S16: Left: Exponential decay of absorbance at 477 nm of **1**$_{\mathrm{VHF}}$ to **1**$_{\mathrm{DHA}}$ in acetonitrile at 35 °C ($t_{1/2}$=1851 min). Right: Spectral evolution during thermal back- reaction of **1** in acetonitrile at 35 °C.

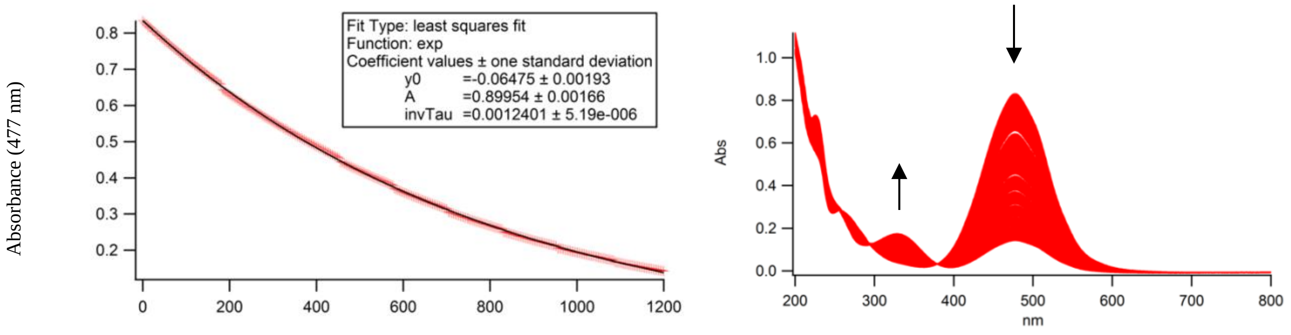

Figure S17: Left: Exponential decay of absorbance at 476 nm of **1**$_{\mathrm{VHF}}$ to **1**$_{\mathrm{DHA}}$ in acetonitrile at 45 °C ($t_{1/2}$=559 min). Right: Spectral evolution during thermal back- reaction of **1** in acetonitrile at 45 °C.

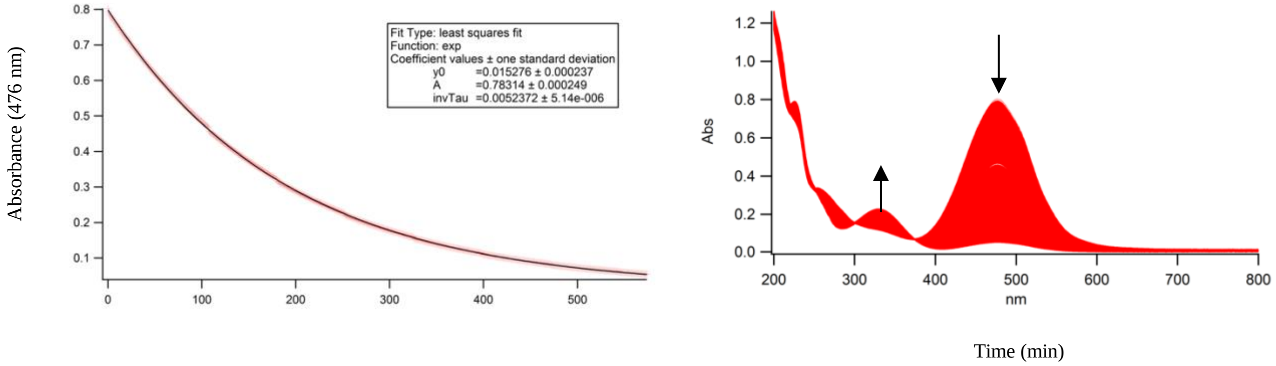

Figure S18: Left: Exponential decay of absorbance at 476 nm of **1**$_{\mathrm{VHF}}$ to **1**$_{\mathrm{DHA}}$ in acetonitrile at 55 °C ($t_{1/2}$=132 min). Right: Spectral evolution during thermal back- reaction of **1** in acetonitrile at 55 °C.

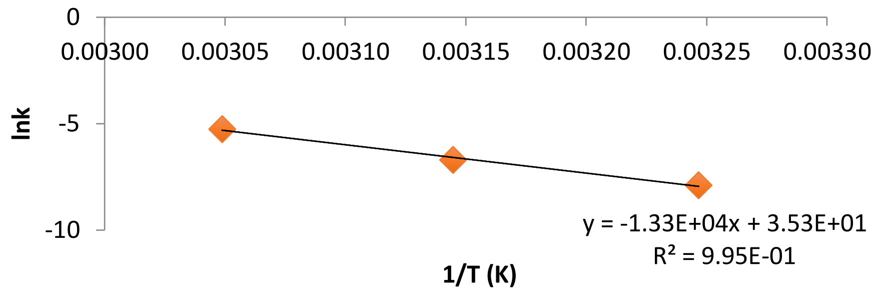

Figure S19: Arrhenius plot for the **1**$_{\text{VHF}}$ to **1**$_{\text{DHA}}$ conversion.

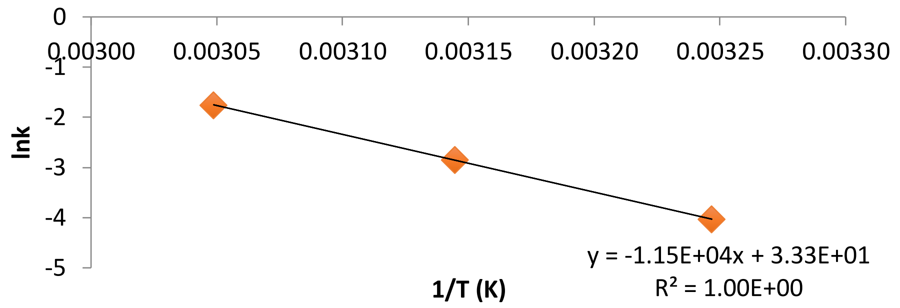

Figure S20: Arrhenius plot for the ***meta*-VHF-Ph-I** to ***meta*-DHA-Ph-I** conversion.

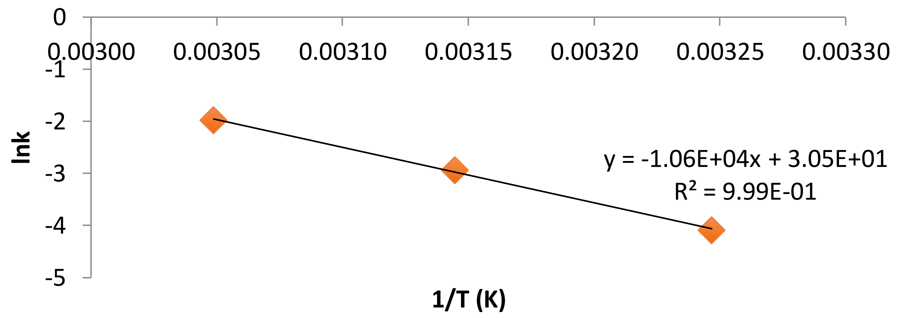

Figure S21: Arrhenius plot for the ***para*-VHF-Ph-I** to ***para*-DHA-Ph-I** conversion.

Compound **2**

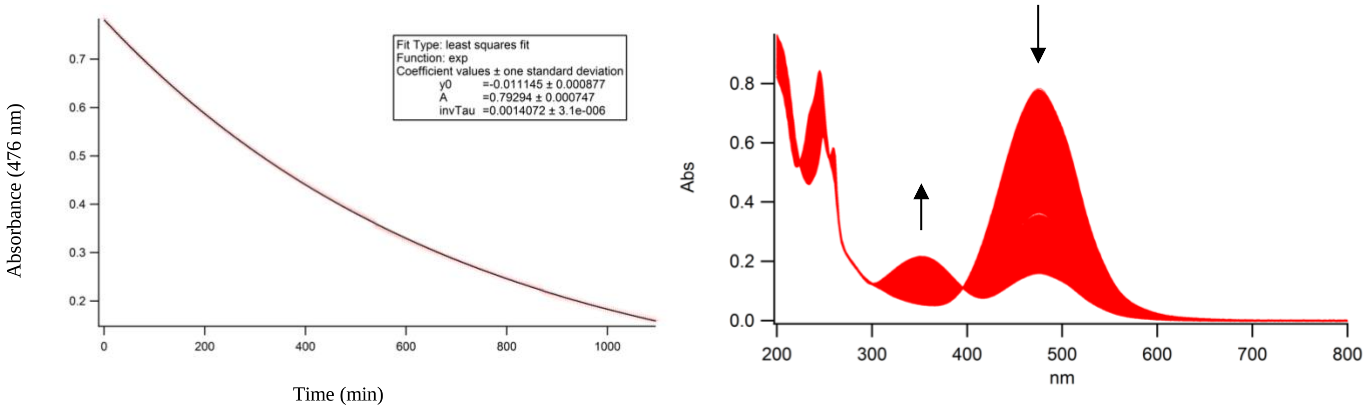

Figure S22: Left: Exponential decay of absorbance at 476 nm of $\mathbf{2}_{\mathrm{VHF}}$ to $\mathbf{2}_{\mathrm{DHA}}$ in acetonitrile at 35 °C ($t_{1/2}$= 492 min). Right: Spectral evolution during thermal back- reaction of **2** in acetonitrile at 35 °C.

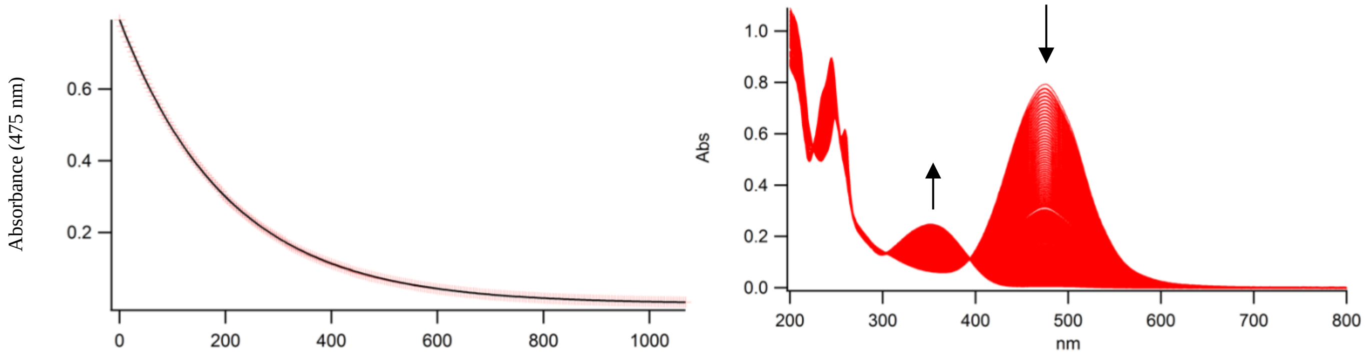

Figure S23: Left: Exponential decay of absorbance at 475 nm of $\mathbf{2}_{\mathrm{VHF}}$ to $\mathbf{2}_{\mathrm{DHA}}$ in acetonitrile at 45 °C ($t_{1/2}$=142 min). Right: Spectral evolution during thermal back- reaction of **2** in acetonitrile at 45 °C.

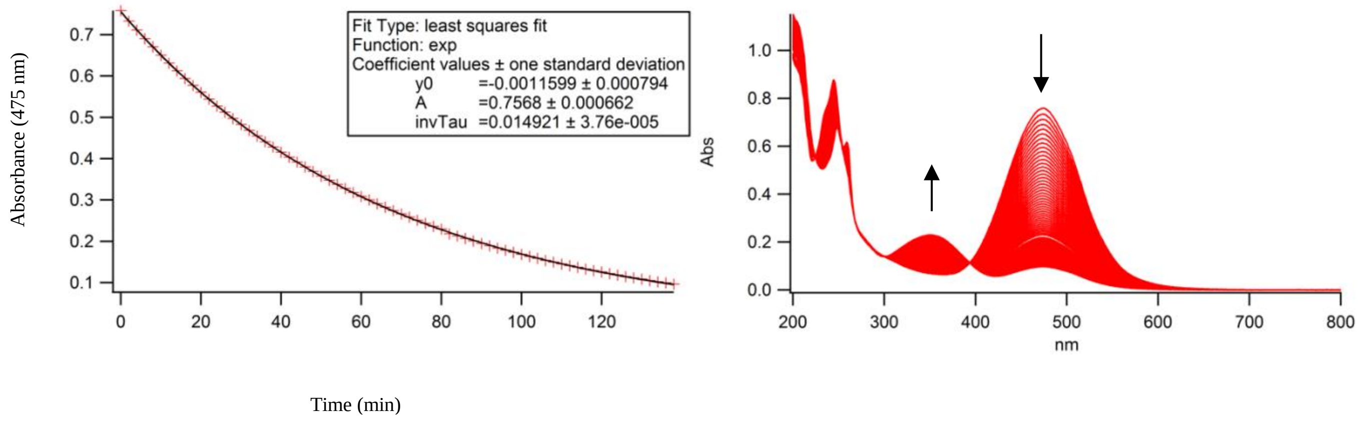

Figure S24: Left: Exponential decay of absorbance at 475 nm of $\mathbf{2}_{\mathrm{VHF}}$ to $\mathbf{2}_{\mathrm{DHA}}$ in acetonitrile at 55 °C ($t_{1/2}$= 46 min). Right: Spectral evolution during thermal back- reaction of **2** in acetonitrile at 55 °C.

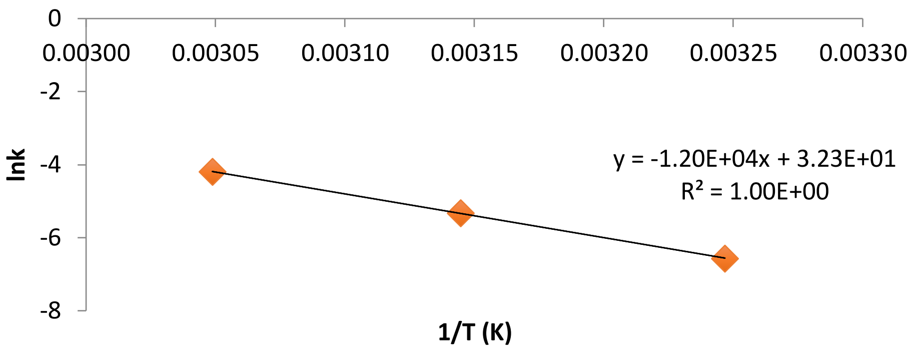

Figure S25: Arrhenius plot for the **2**VHF to **2**DHA conversion.

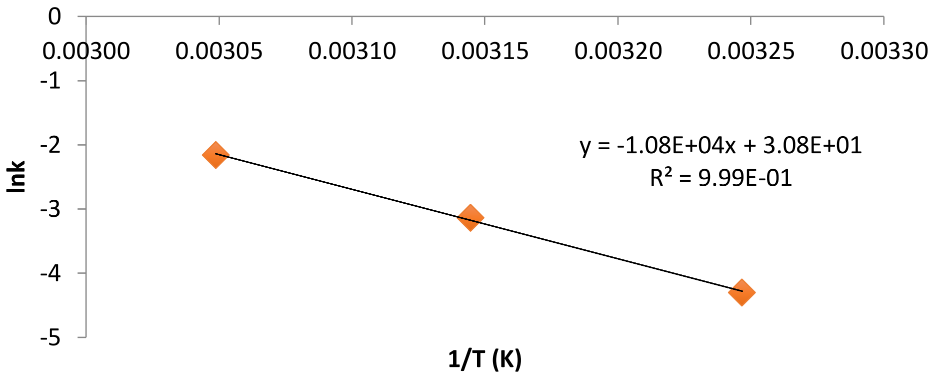

Figure S26: Arrhenius plot for the ***meta*-VHF-Ph-CC-TMS** to ***meta*-DHA-Ph-CC-TMS** conversion.

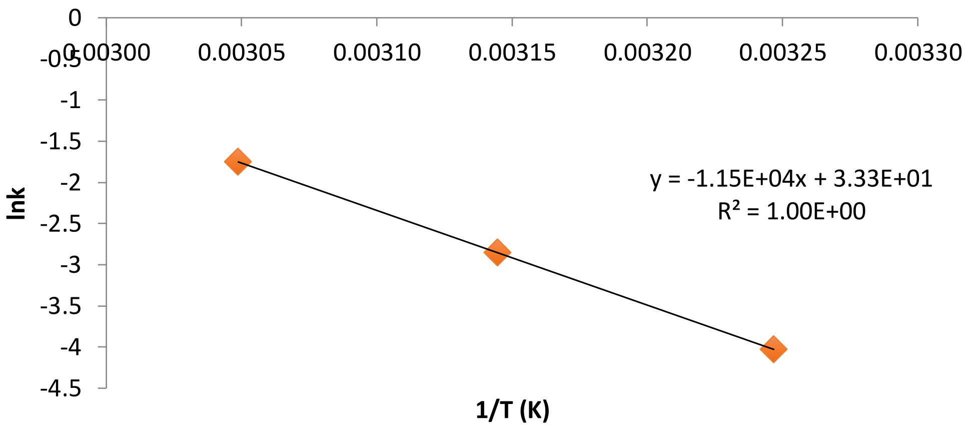

Figure S27: Arrhenius plot for the ***para*-VHF-CC-TMS** to ***para*-DHA-Ph-CC-TMS** conversion.

Compound **3**

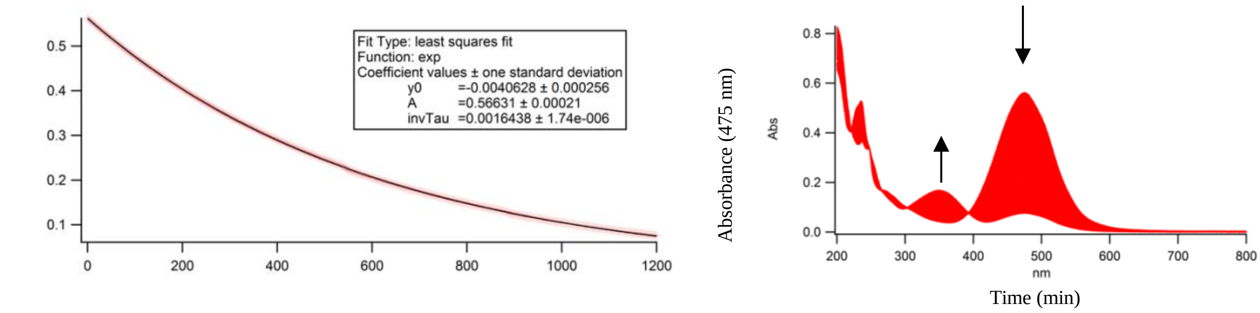

Figure S28: Left: Exponential decay of absorbance at 475 nm of $\mathbf{3}_{VHF}$ to $\mathbf{3}_{DHA}$ in acetonitrile at 35 °C ($t_{1/2}$= 422 min). Right: Spectral evolution during thermal back- reaction of **3** in acetonitrile at 35 °C.

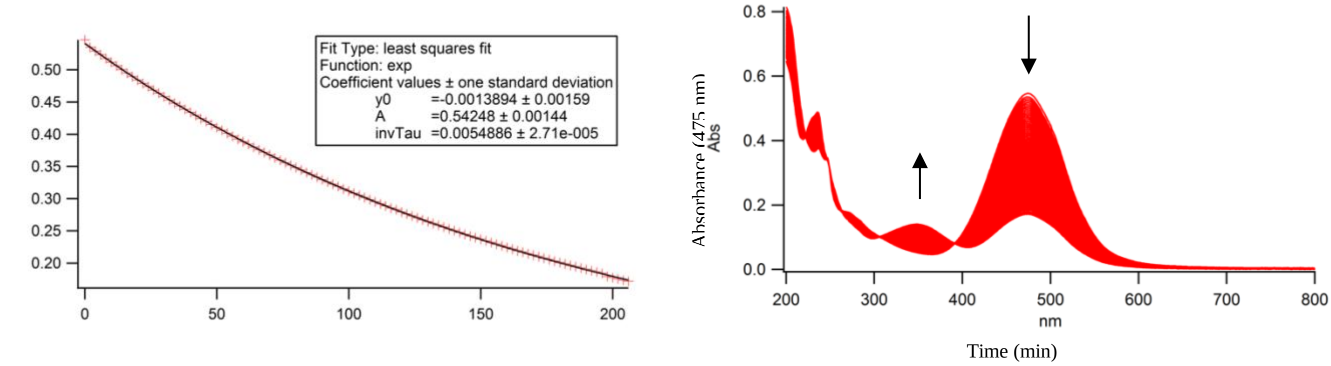

Figure S29: Left: Exponential decay of absorbance at 475 nm of $\mathbf{3}_{VHF}$ to $\mathbf{3}_{DHA}$ in acetonitrile at 45 °C ($t_{1/2}$= 126 min). Right: Spectral evolution during thermal back- reaction of **3** in acetonitrile at 45 °C.

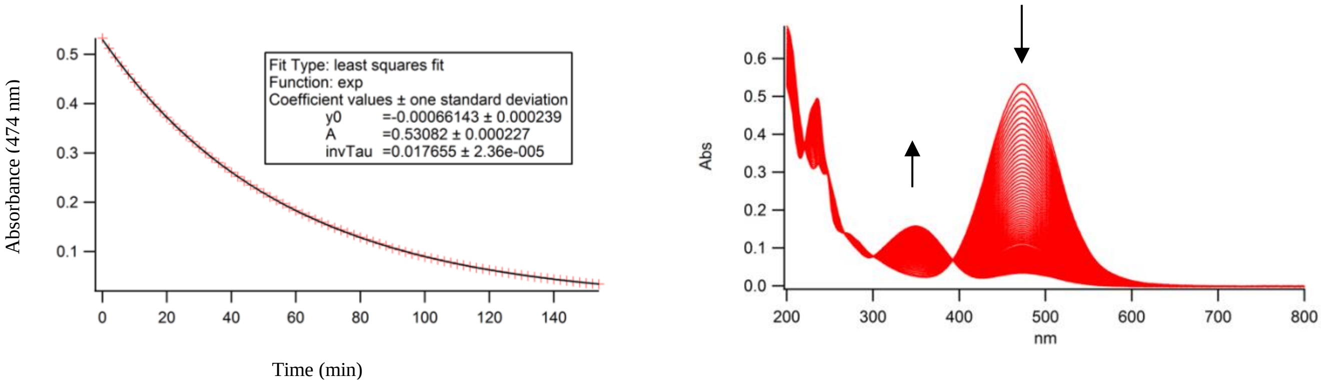

Figure S30: Left: Exponential decay of absorbance at 474 nm of $\mathbf{3}_{VHF}$ to $\mathbf{3}_{DHA}$ in acetonitrile at 55 °C ($t_{1/2}$= 39 min). Right: Spectral evolution during thermal back- reaction of **3** in acetonitrile at 55 °C.

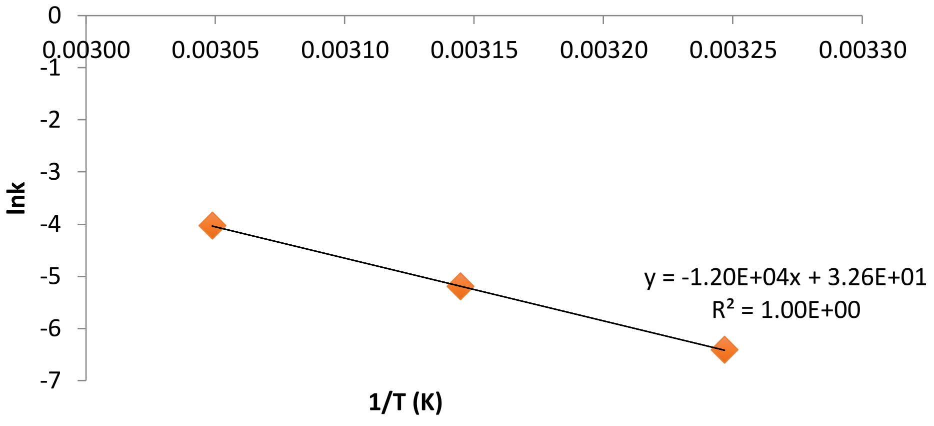

Figure S31: Arrhenius plot for the **3**VHF to **3**DHA conversion.

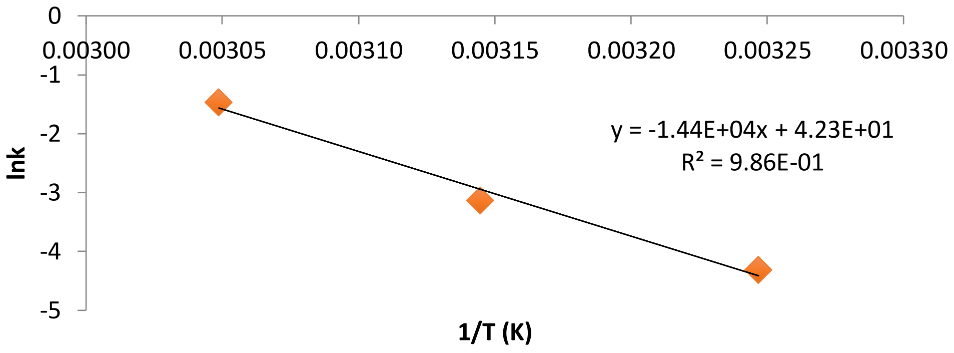

Figure S32: Arrhenius plot for the ***meta*-VHF-Ph-CC-H** to ***meta*-DHA-Ph-CC-H** conversion.

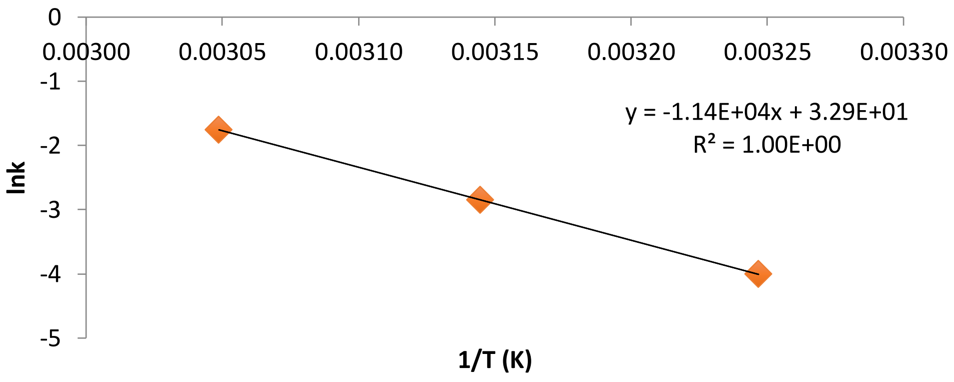

Figure S33: Arrhenius plot for the ***para*-VHF-Ph-CC-H** to ***para*-DHA-Ph-CC-H** conversion.

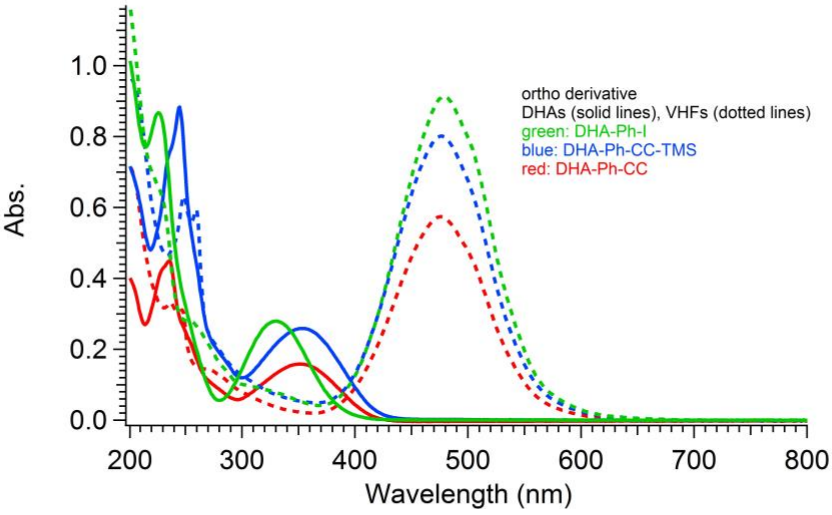

Figure S34: UV-Vis absorption spectra in MeCN of DHAs (solid line) and VHFs (dotted line) for *ortho* compounds **1** (3.3×10^{-5} M, green), **2** (2.6×10^{-5} M, red) and **3** (1.6×10^{-5} M, blue).

Compound **4**

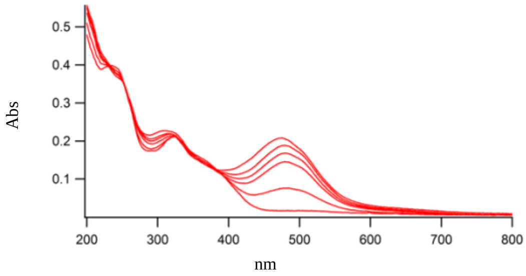

Figure S35: Spectral evolution during ring-opening of **4** in acetonitrile at 25 °C.

Compound **5**

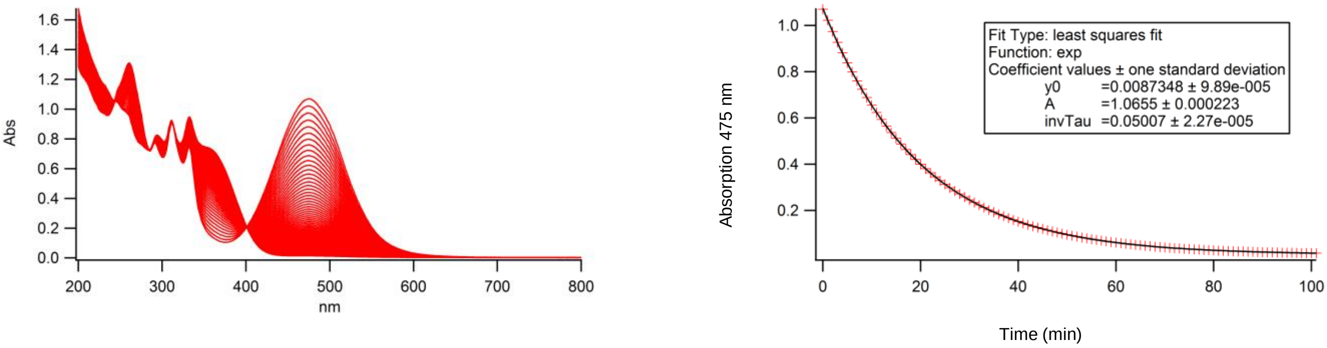

Figure S36: Left: Spectral evolution during thermal back- reaction of **5** in acetonitrile at 45 °C. Right: Exponential decay of absorbance at 474 nm of **5**$_{VHF}$ to **5**$_{DHA}$ in acetonitrile at 45 °C.

Compound **6**

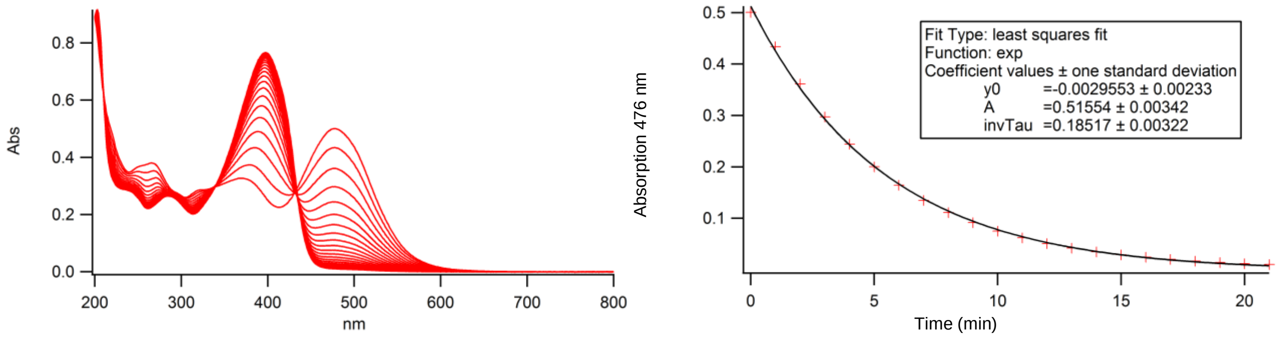

Figure S37: Left: Spectral evolution during thermal back- reaction of **6** in acetonitrile at 55 °C. Top right: Exponential decay of absorbance at 474 nm of **6**VHF to **6**DHA in acetonitrile at 55 °C.

Photoisomerization quantum yields:

<u>Determination of photon flux</u>

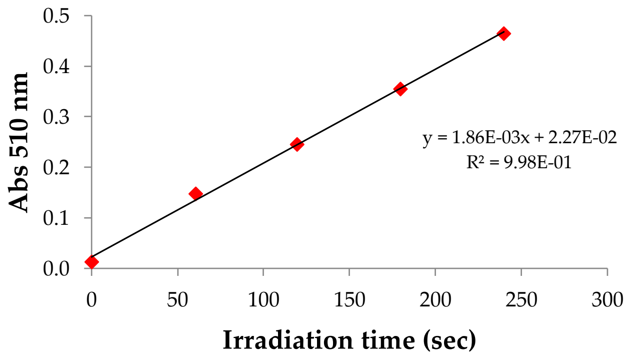

Figure S38: Absorbance of ferrioxalate at 510 nm.

In order to measure the quantum yield, the LED lamp (365 nm) was turned down to an intensity of 30%, and the photon flux was measured in a fixed set-up.

Photon flux: 2.04 x 10^{-8} mol s^{-1}

Determination of quantum yield in acetonitrile

Compound **1**

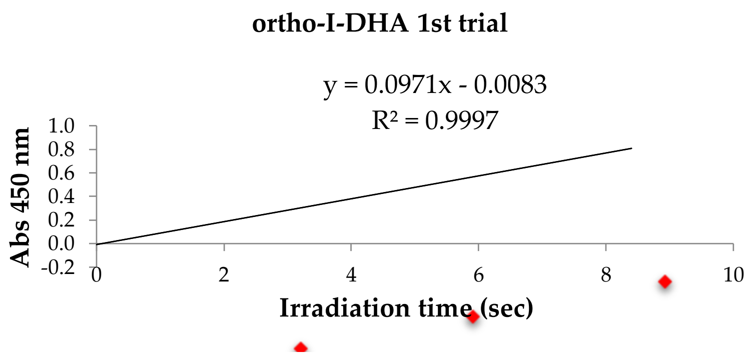

Figure S39. Absorbance of $\mathbf{1}_{VHF}$ (first sample) *vs* irradiation time during irradiation at 365 nm.

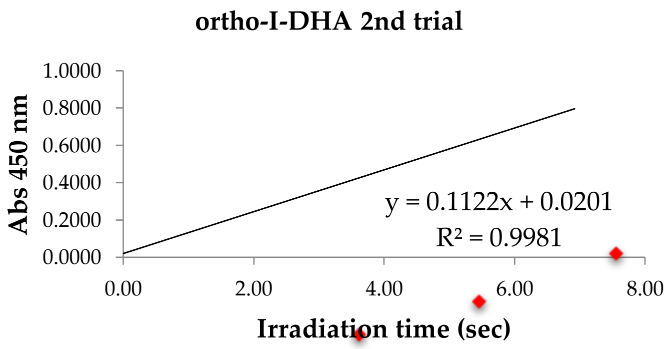

Figure S40. Absorbance of $\mathbf{1}_{VHF}$ (second sample) *vs* irradiation time during irradiation at 365 nm.

Quantum yield for conversion of $\mathbf{1}_{DHA}$ into $\mathbf{1}_{VHF}$:

Average ϕ= 57.4% ≈ 57%

Compound **2**

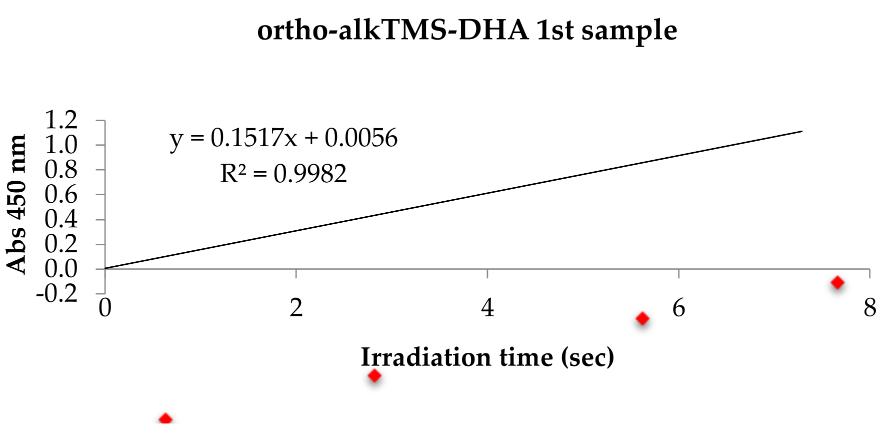

Figure S41. Absorbance of **2**VHF (second sample) *vs* irradiation time during irradiation at 365 nm.

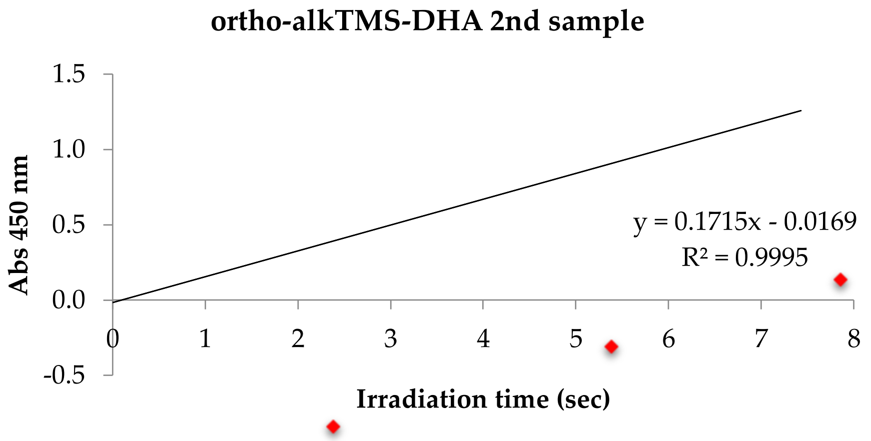

Figure S42. Absorbance of **2**VHF (second sample) *vs* irradiation time during irradiation at 365 nm.

Quantum yield for conversion of **2**DHA into **2**VHF:

Average ϕ= 59.4% ≈ 59%

Compound **3**

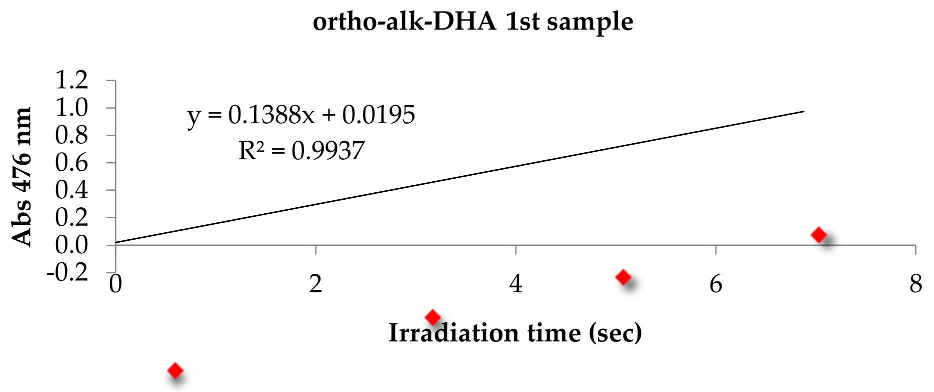

Figure S43. Absorbance of **3**VHF (second sample) *vs* irradiation time during irradiation at 365 nm.

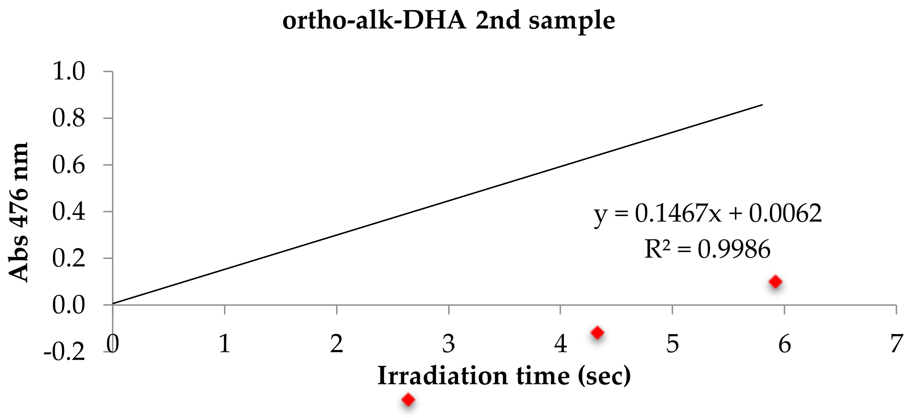

Figure S44. Absorbance of **3**VHF (second sample) *vs* irradiation time during irradiation at 365 nm.

Quantum yield for conversion of **3**DHA into **3**VHF:

Average ϕ= 67.15% ≈ 67%
